# Heterozygosity-Rich Regions in Canine Genome: Can They Serve as Indicators of Balancing Selection?

**DOI:** 10.3390/ani15040612

**Published:** 2025-02-19

**Authors:** Adrián Halvoník, Nina Moravčíková, Luboš Vostrý, Hana Vostra-Vydrova, Gábor Mészáros, Eymen Demir, Monika Chalupková, Radovan Kasarda

**Affiliations:** 1 Institute of Nutrition and Genomics, Slovak University of Agriculture in Nitra, Tr. A. Hlinku 2, 94976 Nitra, Slovakia; xchalupkovam@uniag.sk (M.C.); radovan.kasarda@uniag.sk (R.K.); 2 Department of Genetics and Breeding, Czech University of Life Sciences Prague, Kamýcká 129, Praha-Suchdol, 165 00 Prague, Czech Republic; vostry@af.czu.cz (L.V.); vostrah@af.czu.cz (H.V.-V.); 3 Institute of Livestock Sciences, BOKU University, Gregor-Mendel-Straße 33, 1180 Vienna, Austria; gabor.meszaros@boku.ac.at; 4 Department of Animal Science, Faculty of Agriculture, Akdeniz University, Antalya 07070, Türkiye; eymendemir@akdeniz.edu.tr

**Keywords:** dog, genetic diversity, heterozygosity, runs of homozygosity

## Abstract

Compared to the negative effect of directional selection on genetic diversity, balancing selection acts oppositely and maintains variability across the genome. This study aims to articulate whether balancing selection leads to heterozygosity-rich region islands (HRRIs) forming in the canine genome by investigating 1000 animals belonging to 50 dog breeds via 153,733 autosomal SNPs. A consecutive SNP-based approach was used to identify heterozygosity-rich regions (HRRs). Signals of balancing selection in the genome of studied breeds were then assessed with Tajima’s D statistics. A total of 72,062 HRRs with an average length of 324 kb were detected to be unevenly distributed across the genome. A total of 509 and 450 genomic regions were classified as HRRIs and balancing selection signals, respectively. Although the genome-wide distributions of HRRIs varied across breeds, several HRRIs were found in the same locations across multiple breeds. A total of 109 genomic regions were classified as both HRRIs and signals of balancing selection. Even though the genomic coordinates of HRRIs and balancing selection signals did not fully overlap across all genomic regions, balancing selection may play a significant role in maintaining diversity in regions associated with various cancer diseases, immune response, and bone, skin, and cartilage tissue development.

## Simple Summary

This study identifies heterozygosity-rich regions (HRRs) and HRR islands (HRRIs) in the dog genome and investigates their potential relationship with balancing selection, a form of natural selection that maintains genetic variability. Our research confirmed the formation of HRRIs across the whole genome, with multiple overlaps among different breeds. Also, our findings suggest that these regions contribute to maintaining health and overall fitness in dog populations. Functional annotation of genes within HRRIs suggests roles in cancer, immune response, and tissue development. This could significantly impact breeding practices, potentially leading to healthier and more resilient dogs. Furthermore, understanding these genetic regions can aid in identifying genes linked to diseases, thereby improving veterinary care and treatment strategies.

## 1. Introduction

The classic paradigm in modern evolutionary genetics theory states that any variation arising in a population due to mutation or migration is either removed by purifying selection or brought to fixation by positive selection [1]. However, this claim contrasts with the concept of balancing selection, which refers to any form of natural selection that maintains genetic diversity within populations. The basic principle underlying balancing selection assumes that heterozygosity is widespread and persists in populations due to the advantage of heterozygotes. Although balancing selection footprints have been detected in many species, they have been found near only a limited number of genes, leading to the conclusion that balancing selection is an exception rather than a rule at the genome level. Well-known examples include the major histocompatibility complex (MHC) genes in vertebrates [2,3,4], plant self-incompatibility genes [5], and pathogen resistance genes [6]. Understanding the benefits of balanced allele frequencies in adaptive traits, such as immune response or ability with respect to novel challenges or environments, highlights the importance of maintaining adequate levels of genetic diversity in populations affected by directional selection [7].

The low power and high incidence of false-positives in current statistical approaches to detect observed variations in molecular data have raised the question of whether balancing selection is rare or just difficult to detect [8]. Genome-wide heterozygosity-rich regions (HRRs), on the other hand, are of significant potential to obtain deeper knowledge regarding the effect of balancing selection. As initially mentioned by Williams et al. [9], HRRs are defined as genomic regions with high heterozygosity, whereas they could be incorrectly referred to as runs of heterozygosity in many cases [10]. While in runs of homozygosity (ROH), all base pairs between genotyped SNPs are considered homozygous, the non-genotyped base pairs between genotyped heterozygous calls are not necessarily all heterozygous. Up to now, several studies have observed the non-random HRR distribution and the formation of HRR islands (HRRIs) in the genomes of cattle [11], sheep [12], or goats [13]. If a genomic region consistently exhibits high variability in a substantial proportion of the population, it suggests the presence of specific underlying patterns. The most frequently discussed hypothesis is that HRRIs arise due to balancing selection. Fabbri et al. [14] reported the abundance of HRRIs near genes linked to fertility, survival, and immune response in numerous cattle breeds, where these traits are often associated with balancing selection. However, one thing remains unknown. While the MHC complex, affected by balancing selection, remains highly variable across species and breeds, this is not the case for HRRIs. Although multiple breeds of the same species sometimes share HRRIs, a large proportion of HRRIs appear to be distributed randomly without common genetic patterns between breeds. For example, Selli et al. [12] studied the occurrence of HRRIs in various sheep breeds, and out of a total of 115 HRRIs detected, only 17 HRRIs were found to occur in the same genomic region across breeds. If we compared studies conducted on cattle [10,11], no overlap was observed in the detected HRRIs among the examined breeds.

Detection of balancing selection footprints in the genome is often based on approaches assessing diversity level around locus of interest (Hudson–Kreitman–Agaudé test, Likelihood Test T1), excess of common polymorphisms (Tajima’s D, Fu and Li’s F, Fu and Li’s D), genetic differentiation between populations (*F_ST_*), or linkage disequilibrium (integrated extended haplotype homozygosity) [8]. The most widely preferred approach seems to be Tajima’s D test, which distinguishes between DNA sequences evolving randomly and those undergoing non-random processes. A positive D value indicates balancing selection, reflecting an abundance of intermediate allele frequencies. Conversely, a negative D value indicates the fixation of rare alleles, a signal of positive selection [15,16].

To the best of our knowledge, no studies have been reported that characterise HRRs in the canine genome. Thus, the main aims of this paper were to detect HRRs and HRRIs in multiple dog breeds and perform functional annotation of genes localised in HRRIs. Additionally, we aimed to investigate whether similar purposes, high genetic connectedness, or similar geographical origin of breeds contribute to HRRI formation in shared genomic regions. Finally, we explored if the HRRIs arise as a consequence of balancing selection and can be used to detect its footprints.

## 2. Materials and Methods

### 2.1. Source of Genomic Data and SNP Pruning

Two different sources of genomic data were used in this study: the authors’ own data for two Slovak national dog breeds obtained using genotyping of 74 animals by the CanineHD 230 k BeadChip and genomic data for 3776 animals genotyped by a semicustom Illumina SNP array belonging to 48 dog breeds retrieved from a study by Shannon et al. [17]. A list of all breeds with additional information (number of animals, geographical origin, FCI group and section) is given in Table S1. Across both databases, 153,733 autosomal SNPs with known physical positions according to current dog genome assembly (ROS_Cfam 1.0) were found to be common. Two types of data quality control, depending on the type of analysis, were performed by PLINK 1.9 [18]. For HRR and ROH detection, only genotype and individual call rates were controlled (a minimum call rate set to 0.10; QC1). In the case of Tajima’s D and *F_ST_*, additional control of minor allele frequency with a minimum set to 0.01 was done (QC2). To avoid using closely related animals and to minimise the negative effect of unbalanced sample size across breeds, 20 animals per breed were selected based on the identity-by-descent (IBD) matrix calculated by PLINK 1.9 [18] following the Food and Agriculture Organization of the United Nations (FAO) recommendation for characterisation of animal genetic resources [19]. After SNP pruning, the final dataset included information about 144,407 (QC1) resp. 143,616 (QC2) autosomal SNPs for 1000 animals of 50 breeds.

### 2.2. Detection of HRRs and ROH Islands and Tajima’s D Calculation

The distribution of heterozygosity-rich regions in the autosomal genome of each breed was tested by the consecutive SNP-based approach and the R package detectRUNS [20]. The following parameters were used for HRR detection: a minimum of 20 SNPs in HRRs, a minimum length for each HRR of 50 kb and a maximum gap of 50 kb. In HRRs, three homozygous and one missing SNP were allowed. In the next step, observed heterozygosity (*H_o_*) for each individual was calculated by PLINK 1.9 [18] to quantify if there was a statistically significant correlation between the number and proportion of the genome covered by HRRs and overall heterozygosity across breeds. The R package GGally [21] was used to calculate the Pearson correlation coefficient. HRR islands were then identified based on the frequency of SNPs shared in HRRs by animals separately for each breed. The threshold value defining HRRIs was set to the top 0.1 of the percentile of a frequency of shared SNPs in HRRs, with each HRRI containing at least 2 SNPs.

For the detection of ROH, a model-based approach in the RZooRoH [22,23,24] with multiple homozygous-by-descent (HBD) classes (R = 2, 4, 8, 16, 32, 64) and one non-HBD class (R = 128) was used. The model parameters were set according to the recommendations provided by Bertrand et al. [22]. For other parameters, the default values of the RZooRoH package were used [22,23,24]. ROH islands (ROHI) were defined similarly to HRRIs, i.e., based on a minimum frequency of SNPs shared in ROH by animals (top 0.1%) within each breed. Each ROHI had to consist of at least 2 SNPs. This study focused solely on ROHIs potentially occurring at the same genomic coordinates as HRRIs. Only ROHIs overlapping with detected HRRIs were included in the visualisation, allowing us to assess whether regions of high variability in one breed could be strictly homozygous in another.

Tajima’s D statistics [25] implemented in the R package SnpR [26] were used to quantify the effect of balancing selection on the genome. D values were calculated for the whole genome of each breed separately using a non-overlapping sliding window method, with the window length set to 250 kb. Balancing selection signals were defined separately for each breed, with the threshold value set to the top 0.1% of windows showing the highest positive D values.

The common patterns in the formation of HRRIs and balancing selection signals across genomes of studied breeds were analysed by one-way analysis of variance (ANOVA). The degree of HRRIs or selection signals overlap in specific chromosomal regions across breeds was coded numerically based on chromosomal location and the number of breeds that shared such a region in their genome. We assumed that the main factors contributing to the formation of shared signals by breeds include the geographical origin, degree of genetic connectedness, and purpose of studied breeds. Breeds were grouped by geographical origin into 14 categories reflecting their country of origin. Genetic connectedness was assessed using 11 groups derived from Wright’s *F_ST_* matrix calculated by the R package StAMPP [27] and neighbour-net analysis (Figure S1) in SplitsTree [28]. Breed purpose was classified according to FCI groups and sections, with 10 groups derived from the FCI group and 11 based on the FCI section. The classification of breeds according to these factors is shown in Table S1.

### 2.3. Gene Annotation and Functional Enrichment Analyses

The Ensembl Biomart [29] was used to compile a list of genes located in genomic regions with significant overlaps of HRRIs or balancing selection signals across breeds. The significance of overlaps (*p*-value < 0.05) was determined for each approach separately by calculating the Z-score that showed a minimum required overlap in seven breeds (so-called hot spots of HRRIs or balancing selection signals). The identified genes were then subjected to Functional Annotation, Gene Ontology (GO), and KEGG pathway enrichment analyses using the web-based tool DAVID v6.8 [30,31], with significance for enriched biological processes set at *p*-value < 0.05.

## 3. Results

### 3.1. HRR Detection and Observed Heterozygosity

Across all breeds, 72,062 HRRs with an average length of 324 kb were detected. The average percentage of the genome covered by HRRs per individual was 0.011%. The distribution of these segments was uneven among breeds and chromosomes, whereas individuals within breeds exhibited non-significant variance in the number and percentage of genome covered by HRRs. The lowest number of HRRs (606) and percentage of the genome covered by them (0.004) were observed in Basenji, whereas the highest number of HRRs (3523) and percentage of the genome covered by them (0.026) were found in Boxer. Genome-wide, chromosomes 1 and 22 harboured the highest HRR number (4173 and 4053), while chromosomes 38 and 35 carried the lowest amount of HRRs (71 and 270).

**Figure 1:**
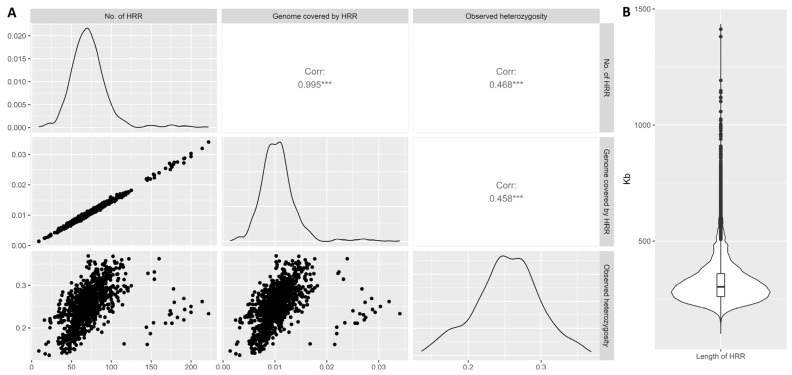
Pairwise Pearson correlations (*** indicates *p* < 0.001) between *H_o_*, percentage of genome covered by HRRs, and number of HRRs (**A**) and violin plot showing variability in the length of detected HRRs (**B**).

The values of *H_o_* ranged from 0.171 (Bull Terrier) to 0.328 (Jack Russell Terrier), with an average of 0.254. As shown in Figure 1A, *H_o_* was significantly correlated with both the HRR number (*r*^2^ = 0.47) and the percentage of the genome covered by HRRs (*r*^2^ = 0.46), indicating that individuals with higher *H_o_* carried more HRRs and HRRs covered a higher proportion of their genome. The highest correlation was observed between the number of HRRs and the percentage of genome covered by them (*r*^2^ = 0.995), likely due to the relatively low variability in HRR lengths, as illustrated in Figure 1B. Detailed information about detected HRRs and *H_o_* per each breed and chromosome is given in the Supplementary Materials (Tables S2 and S4).

### 3.2. HRR Islands and Gene Annotation

**Figure 2:**
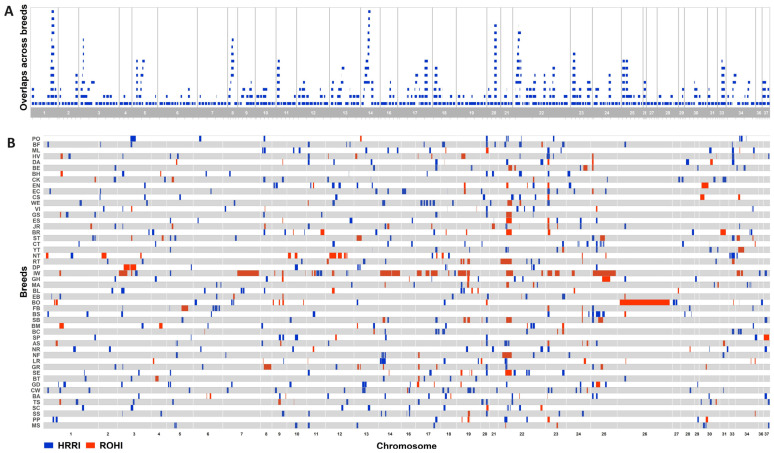
Distribution of HRRIs per chromosome in the whole population (**A**) and distribution of HRRIs (blue) and ROHIs (red) per breed (**B**). The gaps between visualised regions within and between chromosomes do not correspond to the real distance.

The average cut-off for shared SNPs in HRRs was 48.72%, ranging from 35% in the Basenji to 65% in the Belgian Tervuren. Based on these thresholds, 509 HRRIs containing 4982 unique SNPs were identified across all breeds. Identified HRRIs had an average length of 259 kb and included 19,819 SNPs. As shown in Figure 2A, HRRIs were unevenly distributed across the genomes of the studied breeds. Despite that, several “hot spots” where HRRIs accumulated in the same genomic regions across multiple breeds were identified on chromosomes 1, 3, 5, 8, 11, 14, 17, 20, 22, 23, 25, and 33. The genomic coordinates of these hot spots, along with annotated genes, are shown in Table 1. On the other hand, chromosomes 15, 32, 35, and 38 did not harbour any HRRIs. A detailed description of the identified HRRIs is given in Tables S2 and S5.

The next step was to assess whether breeds with HRRIs in the same genomic regions shared common characteristics, such as geographical origin. For example, if breeds with the same origin exhibit HRRIs in the same genomic regions, it would suggest that this factor could significantly influence their formation. The effects of three main factors—breed purpose, genetic connectedness, and geographic origin—were evaluated using one-way ANOVA. The results indicated that all of the tested factors affected the HRRI distribution across breeds only non-significantly (*p* < 0.05).

In this study, the occurrences of ROHIs were tested to explore whether regions that exhibit high variability in one breed might be strictly homozygous in another. Of the 985 ROHIs identified in the studied breeds, 242 overlapped with HRRI genomic coordinates. Figure 2B shows all identified HRRIs (in blue) alongside overlapping ROHIs (in red). Further analysis of ROHI distribution revealed that ROHIs also occurred in genomic regions with HRRI accumulation across multiple breeds. For example, on chromosomes 1 and 14, where the highest proportion of breeds harboured HRRIs, certain breeds, such as the Jack Russell Terrier (chromosome 1) and the Irish Wolfhound, Saint Bernard, Golden Retriever, and Mastiff (chromosome 14), exhibited ROHIs. This confirms that genomic regions highly heterozygous in one breed can be strictly homozygous in another breed.

Based on the assumption that the accumulation of HRRIs in certain genomic regions of the evaluated breeds is driven by the function of genes controlling specific traits, we focused only on the description of genes in HRRI hot spots. A total of 116 identified protein-coding genes located directly in the hot spots area (Table 1) were annotated with six biological processes, six molecular functions, and two cellular components (Table S7). The most significantly enriched GO term was GO:0005516~calmodulin binding (*p* < 0.001), involving five genes (*SLC24A4*, *IQCF3*, *IQCF6*, *ENSCAFG00845018631*, *ENSCAFG00845018549*) located on chromosomes 8 and 20. The process with the highest number of involved genes was KW-0378~Hydrolase, with ten genes (*RAD54L2*, *ST14*, *ADAMTS2*, *LGMN*, *ADAMTS8*, *ENSCAFG00845009907*, *ENSCAFG00845006293*, *ENSCAFG00845009711*, *ENSCAFG00845008741*, *ENSCAFG00845009100*) located on chromosomes 5, 8, 11, 17, 20, and 22.

### 3.3. Signals of Balancing Selection and Gene Annotation

**Figure 3:**
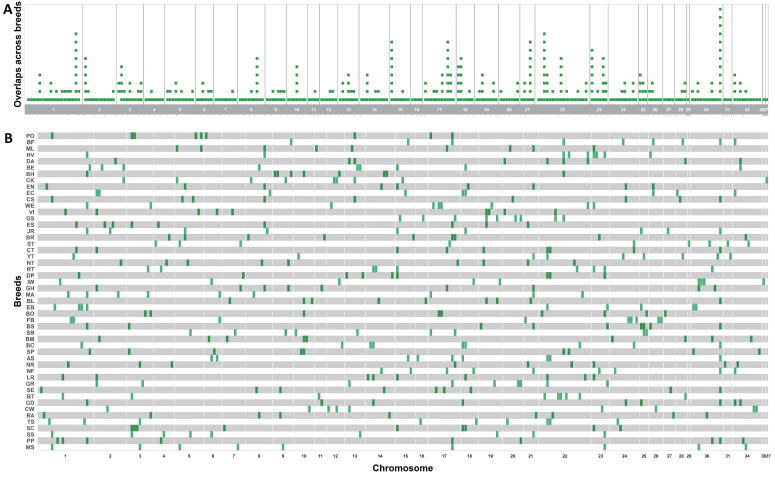
Distribution of balancing selection signals derived from Tajima’s D statistics per chromosome in the whole population (**A**) and per breed (**B**). The gaps between visualised regions within and between chromosomes do not correspond to the real distance.

Based on Tajima’s D statistics, a total of 450 genomic regions across all breeds, with an average D value of 3.655, were classified as signals of balancing selection. The Basenji breed had the lowest average D value (3.492) and proportion of HRRs, while the Boxer breed had the highest average D value (3.807) and proportion of HRRs. Balancing selection signals were unevenly distributed across the genome of breeds (Figure 3A,B), with the highest number on chromosomes 1 (38) and 22 (39), which also exhibited the highest proportions of HRRIs (Tables S2 and S3). Similar to HRRI distribution analysis, chromosomes 32, 35, and 38 did not harbour any signals of balancing selection. As shown in Figure 3A, signals of balancing selection accumulated multiple times in the same genomic regions across breeds, particularly on chromosomes 1, 15, 17, 21, 22, 23, and 30. Complete results of Tajima’s D statistics per breed and chromosome are shown in Tables S3 and S6.

Results from the one-way ANOVA indicated that geographical origin had the most significant effect on the formation of balancing selection signals, with an R^2^ value of 0.110 (*p* < 0.001). Other tested factors (degree of genetic connectedness and breed purpose) had a lower effect, with R^2^ values of 0.084 (*p* < 0.001) for the FCI group, 0.068 (*p* < 0.0005) for the FCI section, and 0.065 (*p* < 0.0005) for genetic distance.

Gene annotation and functional enrichment analyses were performed on genes located directly in the genomic region of overlapping balancing selection signals in at least seven breeds (Table 2). A total of 37 identified protein-coding genes were associated with three biological processes, one molecular function, and one cellular component (Table S8). The most significantly enriched GO term (*p* < 0.005) and also the GO term with the highest number of involved genes was GO:0005829~cytosol. Ten genes (*OSBPL9*, *DENND5A*, *ABHD5*, *NRDC*, *NCOA1*, *PSTPIP1*, *SCAPER*, *SETDB2*, *ENSCAFG00845020170*, *ENSCAFG00845020275*) spread across chromosomes 15, 17, 21, 22, 23, and 30 were involved in this GO term.

**Figure 4:**
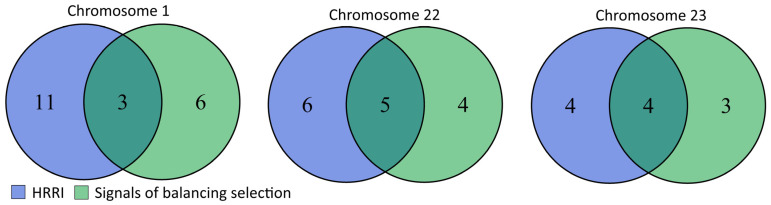
Venn diagrams showing overlaps between balancing selection signatures and HRRIs in regions identified as hot spots of balancing selection signatures and HRRIs.

Comparing the distribution of balancing selection signals and HRRIs across breeds showed a total of 109 overlaps between them (Table S9). Further analysis revealed that these overlaps also occur in regions identified as hot spots of balancing selection signals and HRRIs (Table 1 and Table 2). Among the identified hot spots, this happened on chromosomes 1 (99.750–99.865 Mb), 22 (2–2.145 Mb), and 23 (2.750–2.988 Mb). As illustrated in Figure 4, overlaps occurred on chromosome 1 in three breeds (German Shepherd Dog, Havanese, Weimaraner), on chromosome 22 in five breeds (Australian Shepherd, Dachshund, Golden Retriever, Labrador Retriever, Yorkshire Terrier), and on chromosome 23 in four breeds (Havanese, Maltese, Weimaraner, Nova Scotia Duck Tolling Retriever).

## 4. Discussion

### 4.1. HRR Detection and Observed Heterozygosity

Detecting HRRs requires the predefinition of several parameters, such as minimum SNPs in HRRs, minimum length of HRRs, number of opposites, and missing SNPs, where these parameters directly impact the analysis outcomes. Due to this fact, many studies have tried to address this challenge by testing various settings [12,13,14,32]. However, clear guidelines for defining optimal settings for HRR detection remain lacking. Two methodological approaches, consecutive runs and sliding windows, are currently used to define HRRs. A previous study by Chessari et al. [13] demonstrated that both approaches showed comparable results in HRR detection. Due to its widespread use in previous HRR detection studies [11,14,33,34] and its advantage in enabling better comparison with existing results, only the consecutive runs approach was chosen in this study.

Regarding the predefined settings in HRR analysis, we decided to stick to more lenient criteria where up to three opposite (homozygous) genotypes were allowed in HRRs. This decision was made because previous studies confirmed the occurrence of inbreeding [35,36] and relatively high homozygosity of the canine genome. Also, Ferenčaković et al. [10] emphasised that HRRs need not be strictly heterozygous but should exhibit a higher degree of heterozygosity relative to the rest of the genome. A region in which at least 85% (i.e., 17 out of 20) of all SNPs are heterozygous could still be considered highly variable compared to the rest of the genome. Using stricter criteria would mean that regions still highly heterozygous compared to the rest of the genome would not be considered HRRs. HRRs are known to be short regions [32], so the minimum segment length was set at a relatively low value (50 kb). However, such short segments may not contain enough SNPs, leading to false positives [37,38]. For this reason, the minimum number of SNPs was set to 20, which ensures that each segment contains enough SNPs to avoid false-positive HRR detections [32].

Three key observations were made regarding the detected HRRs. The first concerns the average length of detected HRRs and the length of the longest segment identified. Previous studies have reported significantly higher average lengths of detected HRRs, ranging from 0.45 Mb [12] to 1.67 Mb [39]. In Selli et al. [12], the most common length category for HRRs was between 500 and 750 kb. Similarly, Mulim et al. [40] found that 45.13% of HRRs ranged from 0.5 to 1 Mb, while 41.11% ranged from 1 to 1.5 Mb. However, in this study (Figure 1), the majority of detected HRRs did not exceed 0.5 Mb, revealing a discrepancy with previous findings. This discrepancy was also evident in the length of the longest detected HRR, which in our study reached a maximum of 1.4 Mb in the Boxer breed, with most breeds showing the longest HRRs under 1 Mb. Other studies have reported HRRs longer than 2 Mb [11,33,34,40], and even up to 4 Mb [14,39]. Two factors may account for the differences observed compared to previous findings. First, the genetic backgrounds of the studied species might contribute to variations in HRR formation. Currently, it is not possible to compare our findings with other studies conducted on Canis familiaris or other species from the family Canidae since this is the first study examining the formation of HRRs in this species. The second factor is the density of genomic data analysed. Previous studies on HRR detection have primarily used 50K SNP arrays for genotyping, which may impact HRR length estimates. Supporting this hypothesis, Szmatoła et al. [41] demonstrated that analyses of HRRs in Holstein cattle using 50 K and 700 K SNP arrays showed overestimated HRR lengths and longer HRRs when using the 50 K array. Regarding these findings, there is plenty of evidence on how the density of genomic information influences the results of ROH detection, which is detected by the same methodological approach. Purfield et al. [37] and Ferenčaković et al. [38] claim that the 50 K panel does not provide sufficient information for the estimation of short ROH (1–4 Mb) and has the potential to inflate ROH length. This phenomenon most probably occurs during the process of HRR detection and highly influences its accuracy.

The second observation is related to the low variability in the length of detected HRRs (Figure 1B), which contrasts with previous studies by Mulim et al. [40] and Chen et al. [39]. Low variability in HRR length also explains a high correlation (*r*^2^ = 0.995) between the HRR number and the proportion of the genome covered by HRRs. If an individual carries an HRR in his genome, there is a high probability that the length of this HRR will be consistent.

The last interesting fact is related to the correlation between heterozygosity and the HRR number/proportion of the genome covered by HRRs. As shown in Figure 1, 20 individuals showed a slightly different distribution compared to the whole population. These individuals belong to the Boxer. While this breed has relatively low heterozygosity compared to other breeds in the dataset, their genome contains the highest number of HRRs. Also, as we mentioned earlier, the longest detected HRR belongs to this breed.

Knowledge of the mechanisms influencing HRR number and length across the genome remains limited. Comparison of multiple studies is challenging due to inconsistencies in HRR detection methodological approaches and differences in the density of genomic data used, which significantly influence the results. Furthermore, as discussed in previous studies [9,32], processes such as introgression, admixture, mutations, recombination rate, and chromosomal rearrangements are, together with selection, the driving force behind the formation of HRRs in the genome. In this study, one of the breeds had a significantly higher number of HRRs than the other breeds. However, it is questionable whether these underlying processes affecting genomic architecture would differ in one breed compared to the others to the extent that they would affect the number of HRRs without having the same effect on heterozygosity. Another possibility is the occurrence of admixture or line-crossing that leads to the formation of HRRs and an increase in heterozygosity. It is important to note that admixture or line-crossing may have occurred several generations ago, resulting in the presence of HRRs in the genome [32]. If multiple generations were subsequently subjected to directional selection, this could reduce the overall heterozygosity [7]. However, some HRRs may still be present in the genome from the initial admixture or line-crossing. Further analysis is necessary to fully understand the mechanisms responsible for the formation of HRRs.

### 4.2. HRR and ROH Islands

The uneven distribution of HRRs and HRRIs was described in multiple species [34,40]. This study describes the occurrence of HRRs and HRR islands in the canine genome for the first time. Similar to studies conducted by Selli et al. [12] and Chessari et al. [13], an overlap of HRRIs across multiple breeds of studied species was observed. Based on these, we initially assumed that the factors driving the overlap of HRRIs operate across the entire species in the same way. However, due to factors such as HRR analysis settings and strict threshold criteria for identifying HRRIs, some regions with high heterozygosity, compared to the genome-wide level, were not classified as HRRIs. Moreover, our assumption was also contradicted by the presence of ROHIs in the same genomic regions where HRRI accumulation occurred. In all regions where HRRI accumulation was observed (Table 1), except the region spanning from 2.59 Mb to 2.78 Mb on chromosome 5, some breeds exhibited ROHIs. This was observed not only in the phylogenetically distant breeds but also in the case of closely related breeds such as the Cocker Spaniel and the English Cocker Spaniel. While the English Cocker Spaniel harboured HRRIs on chromosome 23, the Cocker Spaniel showed ROHIs in the same genomic region. The Cocker Spaniel originated from the English Cocker Spaniel, with the division between the two breeds occurring in the mid-19th century when the English Cocker Spaniel was first exported to the USA. Nevertheless, they were still considered the same breed until 1946. In contrast to the English Cocker Spaniel, the purpose of the Cocker Spaniel has remained unchanged (flushing dog), with only minor differences in breeding practices between them, such as selection for a slightly longer frame and a different coat. It is unclear whether these minor changes in breeding practice or the shift to a different environment could cause a significant change in the variability of that genomic region. However, one of the explanations may be that such changes were not caused by artificial (different breeding goals) or natural selection (different environment) but were driven by the founder effect in the separation process of Cocker Spaniel from English Cocker Spaniel.

The functional annotation of protein-coding genes inside HRRIs showed that most of them can be divided into four groups. The first one comprised a group of novel genes (65) with unknown functions. The second one was a group of genes (*CENPP*, *ASPN*, *NOL8*, *EPB41L4A*, *OPCML*, *ST14*, *ZBTB44*, *CPSF2*, *LGMN*, *CHCHD3*, *ZNF512*, *CCDC121*, *MRPL33*, *IQCF6*, *RAD54L2*, *DCAF1*, *TRIM13*, *SPRYD7*, *LHFPL6*, *RCBTB1*) associated with various cancer and tumour diseases [42,43,44,45,46,47,48,49,50,51,52,53,54,55,56,57,58,59,60]. Among these genes, ZBTB44 and ZNF512, which belong to the zinc finger protein (ZNF) gene family, are particularly noteworthy. The presence of ZNF genes in HRRIs of various species has been confirmed by multiple studies [32,34,61]. ZNF genes are not only associated with cancer, but they also play a crucial role in tissue homeostasis, development, and several diseases, such as neurodegeneration, skin disease, and diabetes [62]. These findings suggest a connection between ZNF and heterozygosity that can positively affect various health traits. The third one covered genes associated with bone, skin, and cartilage tissue development or diseases related to these tissues (*ASPN*, *OMD*, *OGN*, *EPB41L4A*, *ADAMTS8*, *ADAMTS2*, *RIN3*, *IQCF3*, *RCBTB1*, *ABHD5*) [45,60,63,64,65,66,67,68,69]. The last group was composed of multiple *U4* and *U6* genes, essential for pre-mRNA splicing of the major class of metazoan nuclear introns [70]. Regarding genes that were not part of the mentioned groups, we need to highlight *MFSD14B*, which is involved in energy homeostasis [71]. Another one is *KPNA3*, which is associated with nuclear protein import and plays a role in Salmonella infection processes [72]. The entire region on chromosome 22, which includes *KPNA3* together with *SETDB2*, *CAB39L*, and *RCBTB1*, was reported as a region under selection pressure containing ROHIs in several hunting dog breeds [73,74]. Mastrangelo et al. [75] identified *SETDB2* as a gene related to pointing behaviour in dogs. This is consistent with our results, as many breeds in our study (Border Terrier, English Setter, Gordon Setter, English Springer Spaniel) with such a background showed the formation of ROHIs in this region.

The genomic region with the *KPNA3* and *SETDB2* genes on chromosome 22, identified as HRRIs in some breeds and ROHIs, mainly in hunting breeds, could help us to explain the overlap between ROHIs and HRRIs. Based on the observed distribution of HRRIs (Table 1) and signals of balancing selection (Table 2), it can be assumed that the *KPNA3* gene is under strong balancing selection pressure, which explains the variability of this region in the studied breeds. However, in the case of hunting breeds, directional selection related to the *SETDB2* gene (pointing behaviour) overwhelmed the effect of balancing selection and led to the formation of ROHIs in the same region in these breeds. Nevertheless, further research, including various phenotypic and genomic data, is necessary to confirm this hypothesis.

### 4.3. Balancing Selection and Its Potential Connection with HRRIs

Balancing selection is often defined as an evolutionary process that maintains genetic diversity within a population by favouring the survival of multiple alleles at a particular gene through heterozygote advantage [76]. Heterozygote advantage, also known as over-dominance, is a phenomenon observed in diploid organisms where heterozygotes are preserved at elevated frequencies within populations. This occurs because heterozygotes exhibit greater fitness compared to either homozygote [8]. The evidence for balancing selection in companion animals and livestock appears relatively limited compared to directional selection. To date, only two studies [15,77] have explored balancing selection in dogs, and neither focused on genome-wide signatures.

Previous studies [14,32] that utilised Tajima’s D statistics to confirm the connection between HRRIs and balancing selection in cattle calculated D values solely in detected HRRIs. In both cases, they confirmed that HRRIs exhibited high positive D values. However, this approach may overlook other genomic regions with higher positive D values. To avoid overlooking these regions, signals of balancing selection were derived purely based on D values, and these genomic coordinates were used for further analysis.

The distribution of balancing selection signals showed a statistically significant impact for each considered factor; however, the R^2^ values were low. This suggests that the distribution of balancing selection signals across the genome may also be influenced by the interaction of selection with other factors such as gene flow, genetic drift, or recombination [8].

When it comes to the comparison of Tajima’s D statistics and HRRIs, theoretically, it can be stated that Tajima’s D statistics and HRRIs measure two aspects of the same process: HRRI detection works with heterozygosity as a result of balancing selection, while significantly positive values of Tajima’s D statistics suggest an excess of intermediate frequency alleles as a consequence of balancing selection. Today, multiple studies have examined HRRIs in different livestock species [9,12,13,34,41]; despite our findings, their connection with balancing selection is still not fully confirmed. On the other hand, Tajima’s D statistics have been widely utilized for the detection of balancing selection [78,79,80,81], but their usage in species under intense directional selection, such as livestock and companion animals, is still limited.

Inside the regions overlapping between balancing selection signals and HRRI hot spots, 17 protein-coding genes were found. Among these genes, 11 were novel genes with unknown functions, while the functions of the remaining six (*MFSD14B*, *KPNA3*, *RCBTB1*, *SETDB2*, *ABHD5*, *U4*) were discussed in the previous chapter related to HRRIs. Regarding genes unique for signals of balancing selection, most of them fall into the same two groups as those in the case of HRRIs. The first was a group of novel genes (7) and the second was a group of genes associated with various cancer and tumour diseases (*TXNDC12*, *NRDC*, *MIR761*, *ITSN2*, *NCOA1*, *SCUBE2*, *DENND5A*, *RCN2*) [82,83,84,85,86,87,88,89]. Genes that did not fit into these categories included *PSTPIP1*, *TMEM41B*, *SCAPER*, and *OSBPL9*. *PSTPIP1* and *TMEM41B* genes were associated with immune responses [90,91]. On the other hand, the *SCAPER* gene was linked to retinitis pigmentosa and intellectual disability [92] and the *OSBPL9* gene was identified as a gene involved in canine hypoadrenocorticism [93]. As discussed in previous studies, balancing selection is a form of natural selection that plays an important role in maintaining genetic diversity, often concerning health and fitness traits, by affecting the frequency of genetic variants associated with these traits. Obtained results support this hypothesis, revealing that a substantial proportion of genes within regions showing signals of balancing selection were functionally annotated with various diseases and immunity-related traits.

## 5. Conclusions

This study focused on detecting HRRs and their potential relationship with balancing selection in the canine genome. The study results confirmed the formation of HRRIs across the whole genome, with multiple overlaps among different breeds. Initially, it was assumed that the regions covered by HRRIs were highly heterozygous across the entire species. However, the presence of ROHIs in these regions in certain breeds contradicted this hypothesis. The genome-wide distribution of HRRIs was non-significantly affected by geographical origin, genetic connectedness, or purpose of studied dog breeds. On the other hand, the genome-wide distribution of balancing selection signals derived from Tajima’s D statistics was significantly affected mainly by the geographical origin of breeds. Similar to HRRIs, detected selection signals overlapped among breeds on specific chromosomes. A comparison of the genomic coordinates of HRRIs and balancing selection signatures revealed that the same genomic region was identified as an HRRI and also as a signal of balancing selection 109 times. This supports the hypothesis that HRRIs may arise in the genome due to balancing selection. Additionally, the analysis of annotated genes suggests that high heterozygosity may positively influence the genetic control of various tumour diseases, as well as the development of bone, skin, and cartilage tissue and immune response.

## Figures and Tables

**Table 1:** Description of HRRI hot spots defined by overlapping HRRIs in at least seven breeds.

Genomic Coordinates	Breeds	Genes
1:99483627-99915987	GD, GS, HV, NT, NR, TS, CT, WE, SC, SE, BT, BS, CW, ST	*CENPP*, *ASPN*, *OMD*, *OGN*, *U6*, *NOL8*, *IARS1*, *SNORA84*, *U4*, *ENSCAFG00845000133*, *ENSCAFG00845000136*, *ENSCAFG00845000140*, *MFSD14B*
3:386718-706891	BS, BL, GD, BH, SS, FB, GR, HV, JR, VI	*EPB41L4A*, *ENSCAFG00845003705*, *ENSCAFG00845003710*, *ENSCAFG00845003718*, *ENSCAFG00845003724*, *ENSCAFG00845003733*
5:2594281-2783066	ES, GD, HV, IW, ST, SE, YT	*OPCML*, *NTM*, *ENSCAFG00845005882*, *ENSCAFG00845005893*, *ENSCAFG00845005901*
	MS, ST, MS, AS, NF, AS, HV	*U6*, *ADAMTS8*, *ST14*, *ZBTB44*, *ENSCAFG00845006246*, *ENSCAFG00845006293*, *ENSCAFG00845006429*
8:1445032-2102164	BM, AS, ML, GS, CK, BR, ML, PO, GS, BL	*CPSF2*, *SLC24A4*, *LGMN*, *RIN3*, *GOLGA5*, *ENSCAFG00845021050*, *ENSCAFG00845021059*, *ENSCAFG00845021071*, *ENSCAFG00845022071*, *ENSCAFG00845022083*, *ENSCAFG00845022228*, *ENSCAFG00845022289*, *ENSCAFG00845022506*
11:2259937-2493519	BF, DA, HV, JR, MS, SE, SS	*ADAMTS2*
14:2948414-4015394	LR, SS, NF, LR, NF, WE, BT, BA, BC, JR, NF, RT, SS, SP	*LRGUK*, *EXOC4*, *CHCHD3*, *ENSCAFG00845015034*
17:21887297-22289388	BE, BC, DA, GS, PP, YT, SB	*ZNF512*, *CCDC121*, *SUPT7L*, *MRPL33*, *ENSCAFG00845008741*, *ENSCAFG00845009100*, *ENSCAFG00845009711*, *ENSCAFG00845009794*, *ENSCAFG00845009819*, *ENSCAFG00845009834*, *ENSCAFG00845009852*, *ENSCAFG00845009882*, *ENSCAFG00845009907*
20:38184737-38519198	GR, AS, BH, BF, GS, PO, SB, SS, SP, SE, TS, VI	*IQCF3*, *IQCF6*, *TEX264*, *GRM2*, *RAD54L2*, *DCAF1*, *ENSCAFG00845018549*, *ENSCAFG00845018631*, *ENSCAFG00845018971*, *ENSCAFG00845019259*, *ENSCAFG00845019283*
22:1719379-2132676	AS, BF, PP, LR, GR, YT, JR, PO, GD, DA, SC	*U6*, *U6*, *TRIM13*, *SPRYD7*, *KPNA3*, *ENSCAFG00845022475*, *ENSCAFG00845022579*, *ENSCAFG00845022562*, *ENSCAFG00845022587*, *ENSCAFG00845022603*, *ENSCAFG00845022619*, *ENSCAFG00845022995*, *ENSCAFG00845023010*, *ENSCAFG00845023073*
22:2180601-2376165	JR, LR, PO, SC, AS, PO, YT	*RCBTB1*, *SETDB2*, *SETDB2*, *CAB39L*
23:2497591-2976833	HV, EC, ML, WE, BS, NR, BH, CW	*ABHD5*, *U4*, *ENSCAFG00845019764*, *ENSCAFG00845019844*, *ENSCAFG00845019895*, *ENSCAFG00845019905*, *ENSCAFG00845019928*, *ENSCAFG00845019943*, *ENSCAFG00845019957*, *ENSCAFG00845019966*, *ENSCAFG00845019972*, *ENSCAFG00845019982*, *ENSCAFG00845020011*, *ENSCAFG00845020052*, *ENSCAFG00845020062*, *ENSCAFG00845020170*, *ENSCAFG00845020203*, *ENSCAFG00845020239*, *ENSCAFG00845020275*
25:1599195-1907645	BA, BS, FB, GD, SB, ST, GR	*COG6*, *LHFPL6*
25:2268042-2548504	AS, BS, CT, DP, ES, YT, BA	*FREM2*, *ENSCAFG00845025825*, *ENSCAFG00845025887*
33:3910692-4140469	NT, BC, DA, HV, ML, ST, BF	*ENSCAFG00845025887*

**Table 2:** Description of overlapping balancing selection signals in at least seven breeds.

Genomic Coordinates	Breeds	Genes
1:99750000-100000000	BH, BR, EB, GD, HV, JR, MA, PP, WE	*MFSD14B*, *ENSCAFG00845000133*, *ENSCAFG00845000136*, *ENSCAFG00845000140*, *ENSCAFG00845000155*, *ENSCAFG00845000164*, *ENSCAFG00845000174*, *ENSCAFG00845000170*, *ENSCAFG00845000180*
15:9500000-9750000	BE, CT, DP, EN, JR, LR, RT, SC	*TXNDC12*, *NRDC*, *MIR761*, *OSBPL9*, *U3*
17:19000000-19250000	AS, BF, BR, ES, GR, MS, PP, PO	*ITSN2*, *NCOA1*
21:33250000-33500000	BR, EN, GH, ML, MA, NF, SS, YT	*SCUBE2*, *DENND5A*, *TMEM41B*
22:2000000-2250000	AS, CT, DA, DP, EB, GR, LR, TS, YT	*KPNA3*, *RCBTB1*, *SETDB2*, *ENSCAFG00845022995*, *ENSCAFG00845023010*, *ENSCAFG00845023073*, *ENSCAFG00845023177*
23:2750000-3000000	HV, LR, ML, NR, RT, SC, WE	*ABHD5*, *U4*, *ENSCAFG00845020052*, *ENSCAFG00845020062*, *ENSCAFG00845020170*, *ENSCAFG00845020203*, *ENSCAFG00845020239*, *ENSCAFG00845020275*
30:39500000-39750000	AS, BF, BC, BR, BL, CT, CS, GD, JR, NF, PP, SE	*PSTPIP1*, *RCN2*, *SCAPER*

## Data Availability

Part of the dataset used in this study originates from research conducted by Shannon et al. [17] and has been deposited in Dryad. The remaining part of the dataset is available from the corresponding author upon reasonable request.

## Supplementary Materials

The following supporting information can be downloaded at https://www.mdpi.com/article/10.3390/ani15040612/s1: Figure S1: Neighbour-joining tree constructed based Wright’s *F_ST_* matrix among studied breeds; Table S1: List of examined breeds with their abbreviation, number of genotyped individuals, country of origin, category based on the degree of genetic distance, and FCI group and section; Table S2: Information about HRRs, HRRIs, and observed heterozygosity per each autosomal chromosome with standard errors in brackets; Table S3: Information about Tajima’s D statistics results per each autosomal chromosome with standard errors in brackets; Table S4: Descriptive statistics for HRRs per each breed and whole metapopulation with standard errors in brackets (standard error for % of genome covered by HRRs is not provided because all values were under three decimal points); Table S5: Information about number, average length, and average number of SNPs of detected HRRIs per each breed and metapopulation with standard errors in brackets; Table S6: Information about Tajima’s D statistics results per each breed and whole metapopulation with standard errors in brackets; Table S7: Results of gene enrichment analysis based on annotated genes within HRR islands detected in at least seven breeds in the same genomic region; Table S8: Results of gene enrichment analysis based on annotated genes within signals of balancing selection detected in at least seven breeds in the same genomic region; Table S9: List of all overlaps between HRRIs and signals of balancing selection.

## Abbreviations

The following abbreviations are used in this manuscript:

ANOVA	Analysis of variance
FAO	Organization of the United Nations
FCI	The Fédération Cynologique Internationale
GO	Gene ontology
HBD	Homozygous-by-descent
*H_o_*	Observed heterozygosity
HRRs	Heterozygosity-rich regions
HRRIs	Heterozygosity-rich region islands
IBD	Identity-by-descent
MHC	Major histocompatibility complex
QC	Quality control
ROH	Runs of homozygosity
ROHIs	Runs of homozygosity islands
SNP	Single nucleotide polymorphism

## References

[1] Booker T.R., Jackson B.C., Keightley P.D. (2017). Detecting Positive Selection in the Genome. BMC Biol..

[2] Aguilar A., Roemer G., Debenham S., Binns M., Garcelon D., Wayne R.K. (2004). High MHC Diversity Maintained by Balancing Selection in an Otherwise Genetically Monomorphic Mammal. Proc. Natl. Acad. Sci. USA.

[3] Slade J.W.G., Watson M.J., MacDougall-Shackleton E.A. (2019). “Balancing” Balancing Selection? Assortative Mating at the Major Histocompatibility Complex despite Molecular Signatures of Balancing Selection. Ecol. Evol..

[4] Moravčíková N., Kasarda R., Vydrova H.V., Vostry L., Karásková B., Candrák J., Halo M. (2024). Genomic Variability of the MHC Region: Empirical Evidence from Five Horse Breeds. Livest. Sci..

[5] Le Veve A., Burghgraeve N., Genete M., Lepers-Blassiau C., Takou M., De Meaux J., Mable B.K., Durand E., Vekemans X., Castric V. (2022). Long-Term Balancing Selection and the Genetic Load Linked to the Self-Incompatibility Locus in *Arabidopsis Halleri* and *A. lyrata*. Mol. Biol. Evol..

[6] Karasov T.L., Kniskern J.M., Gao L., DeYoung B.J., Ding J., Dubiella U., Lastra R.O., Nallu S., Roux F., Innes R.W. (2014). The Long-Term Maintenance of a Resistance Polymorphism through Diffuse Interactions. Nature.

[7] Lacy R.C. (1987). Loss of Genetic Diversity from Managed Populations: Interacting Effects of Drift, Mutation, Immigration, Selection, and Population Subdivision. Conserv. Biol..

[8] Fijarczyk A., Babik W. (2015). Detecting Balancing Selection in Genomes: Limits and Prospects. Mol. Ecol..

[9] Williams J.L., Hall S.J.G., Del Corvo M., Ballingall K.T., Colli L., Ajmone Marsan P., Biscarini F. (2016). Inbreeding and Purging at the Genomic Level: The Chillingham Cattle Reveal Extensive, Non-random SNP Heterozygosity. Anim. Genet..

[10] Ferenčaković M., Banadinović M., Mercvajler M., Khayat-Zadeh N., Mészáros G., Cubric-Curik V., Sölkner J. (2016). Mapping of Heterozygosity Rich Regions in Austrian Pinzgauer Cattle. Acta Agric. Slov..

[11] Biscarini F., Mastrangelo S., Catillo G., Senczuk G., Ciampolini R. (2020). Insights into Genetic Diversity, Runs of Homozygosity and Heterozygosity-Rich Regions in Maremmana Semi-Feral Cattle Using Pedigree and Genomic Data. Animals.

[12] Selli A., Ventura R.V., Fonseca P.A.S., Buzanskas M.E., Andrietta L.T., Balieiro J.C.C., Brito L.F. (2021). Detection and Visualization of Heterozygosity-Rich Regions and Runs of Homozygosity in Worldwide Sheep Populations. Animals.

[13] Chessari G., Criscione A., Marletta D., Crepaldi P., Portolano B., Manunza A., Cesarani A., Biscarini F., Mastrangelo S. (2024). Characterization of Heterozygosity-Rich Regions in Italian and Worldwide Goat Breeds. Sci. Rep..

[14] Fabbri M.C., Tiezzi F., Crovetti A., Maltecca C., Bozzi R. (2024). Investigation of Cosmopolitan and Local Italian Beef Cattle Breeds Uncover Common Patterns of Heterozygosity. Animal.

[15] Cao X., Irwin D.M., Liu Y.-H., Cheng L.-G., Wang L., Wang G.-D., Zhang Y.-P. (2014). Balancing Selection on CDH2 May Be Related to the Behavioral Features of the Belgian Malinois. PLoS ONE.

[16] Nayak S.S., Panigrahi M., Rajawat D., Jain K., Sharma A., Bhushan B., Dutt T. (2024). Unique Footprints of Balancing Selection in Bovine Genome. 3 Biotech.

[17] Shannon L.M., Boyko R.H., Castelhano M., Corey E., Hayward J.J., McLean C., White M.E., Abi Said M., Anita B.A., Bondjengo N.I. (2015). Genetic Structure in Village Dogs Reveals a Central Asian Domestication Origin. Proc. Natl. Acad. Sci. USA.

[18] Chang C.C., Chow C.C., Tellier L.C., Vattikuti S., Purcell S.M., Lee J.J. (2015). Second-Generation PLINK: Rising to the Challenge of Larger and Richer Datasets. Gigascience.

[19] Ajmone-Marsan P., Boettcher P.J., Ginja C., Kantanen J., Lenstra J.A. (2023). Genomic Characterization of Animal Genetic Resources.

[20] Biscarini F., Cozzi P., Gaspa G., Marras G. (2018). DetectRUNS: Detect Runs of Homozygosity and Runs of Heterozygosity in Diploid Genomes. https://cran.r-project.org/web/packages/detectRUNS/vignettes/detectRUNS.vignette.html.

[21] Schloerke B., Crowley J., Cook D. (2018). Package ‘GGally’’. Extension to ‘ggplot2’. https://cran.r-project.org/web/packages/GGally/index.html.

[22] Bertrand A.R., Kadri N.K., Flori L., Gautier M., Druet T. (2019). RZooRoH: An R Package to Characterize Individual Genomic Autozygosity and Identify Homozygous-by-descent Segments. Methods Ecol. Evol..

[23] Druet T., Gautier M. (2022). A Hidden Markov Model to Estimate Homozygous-by-Descent Probabilities Associated with Nested Layers of Ancestors. Theor. Popul. Biol..

[24] Druet T., Gautier M. (2017). A Model-based Approach to Characterize Individual Inbreeding at Both Global and Local Genomic Scales. Mol. Ecol..

[25] Tajima F. (1989). Statistical Method for Testing the Neutral Mutation Hypothesis by DNA Polymorphism. Genetics.

[26] Hemstrom W., Jones M. (2023). SnpR: User Friendly Population Genomics for SNP Data Sets with Categorical Metadata. Mol. Ecol. Resour..

[27] Pembleton L.W., Cogan N.O.I., Forster J.W. (2013). StAMPP: An R Package for Calculation of Genetic Differentiation and Structure of Mixed-ploidy Level Populations. Mol. Ecol. Resour..

[28] Huson D.H. (1998). SplitsTree: Analyzing and Visualizing Evolutionary Data. Bioinformatics.

[29] Kinsella R.J., Kahari A., Haider S., Zamora J., Proctor G., Spudich G., Almeida-King J., Staines D., Derwent P., Kerhornou A. (2011). Ensembl BioMarts: A Hub for Data Retrieval across Taxonomic Space. Database.

[30] Huang D.W., Sherman B.T., Lempicki R.A. (2009). Systematic and Integrative Analysis of Large Gene Lists Using DAVID Bioinformatics Resources. Nat. Protoc..

[31] Sherman B.T., Hao M., Qiu J., Jiao X., Baseler M.W., Lane H.C., Imamichi T., Chang W. (2022). DAVID: A Web Server for Functional Enrichment Analysis and Functional Annotation of Gene Lists (2021 Update). Nucleic Acids Res..

[32] Mulim H.A., Pedrosa V.B., Pinto L.F.B., Tiezzi F., Maltecca C., Schenkel F.S., Brito L.F. (2024). Detection and Evaluation of Parameters Influencing the Identification of Heterozygous-Enriched Regions in Holstein Cattle Based on SNP Chip or Whole-Genome Sequence Data. BMC Genom..

[33] Marras G., Wood B.J., Makanjuola B., Malchiodi K., Peeters P., van As P., Baes C.F., Biscarini F. Characterization of Runs of Homozygosity and Heterozygosity-Rich Regions in a Commercial Turkey (Meleagris Gallopavo) Population. Proceedings of the 11th World Congress of Genetics Applied to Livestock Production.

[34] Ruan D., Yang J., Zhuang Z., Ding R., Huang J., Quan J., Gu T., Hong L., Zheng E., Li Z. (2022). Assessment of Heterozygosity and Genome-Wide Analysis of Heterozygosity Regions in Two Duroc Pig Populations. Front. Genet..

[35] Calboli F.C.F., Sampson J., Fretwell N., Balding D.J. (2008). Population Structure and Inbreeding from Pedigree Analysis of Purebred Dogs. Genetics.

[36] Moravčíková N., Kasarda R., Židek R., Vostrý L., Vostrá-Vydrová H., Vašek J., Čílová D. (2021). Czechoslovakian Wolfdog Genomic Divergence from Its Ancestors Canis Lupus, German Shepherd Dog, and Different Sheepdogs of European Origin. Genes.

[37] Purfield D.C., Berry D.P., McParland S., Bradley D.G. (2012). Runs of Homozygosity and Population History in Cattle. BMC Genet..

[38] Ferenčaković M., Sölkner J., Curik I. (2013). Estimating Autozygosity from High-Throughput Information: Effects of SNP Density and Genotyping Errors. Genet. Sel. Evol..

[39] Chen Z., Zhang Z., Wang Z., Zhang Z., Wang Q., Pan Y. (2022). Heterozygosity and Homozygosity Regions Affect Reproductive Success and the Loss of Reproduction: A Case Study with Litter Traits in Pigs. Comput. Struct. Biotechnol. J..

[40] Mulim H.A., Brito L.F., Pinto L.F.B., Ferraz J.B.S., Grigoletto L., Silva M.R., Pedrosa V.B. (2022). Characterization of Runs of Homozygosity, Heterozygosity-Enriched Regions, and Population Structure in Cattle Populations Selected for Different Breeding Goals. BMC Genom..

[41] Szmatoła T., Gurgul A., Jasielczuk I., Ropka-Molik K. (2024). Comprehensive Analysis of Runs of Homozygosity and Heterozygosity in Holstein Cattle on the Basis of Medium and High Density SNP Panels and Large Population Sample. Ann. Anim. Sci..

[42] Simpson S., Dunning M., de Brot S., Alibhai A., Bailey C., Woodcock C.L., Mestas M., Akhtar S., Jeyapalan J.N., Lothion-Roy J. (2020). Molecular Characterisation of Canine Osteosarcoma in High Risk Breeds. Cancers.

[43] Giantin M., Granato A., Baratto C., Marconato L., Vascellari M., Morello E.M., Vercelli A., Mutinelli F., Dacasto M. (2014). Global Gene Expression Analysis of Canine Cutaneous Mast Cell Tumor: Could Molecular Profiling Be Useful for Subtype Classification and Prognostication?. PLoS ONE.

[44] Vadlamudi S.N. (2024). Detection of Cell-Free Tumor DNA in Liquid Biopsies of Dogs with B Cell Lymphoma: A Biomarker Discovery. Ph.D. Thesis.

[45] Zhang W., Lai R., He X., Liu X., Zhang Y., Yang Z., Yang P., Wang J., Hu K., Yuan X. (2020). Clinical Prognostic Implications of EPB41L4A Expression in Multiple Myeloma. J. Cancer.

[46] Ye F., Zhang S.-F., Xie X., Lu W.-G. (2008). OPCML Gene Promoter Methylation and Gene Expression in Tumor and Stroma Cells of Invasive Cervical Carcinoma. Cancer Investig..

[47] Kauppinen J.M., Kosma V.-M., Soini Y., Sironen R., Nissinen M., Nykopp T.K., Kärjä V., Eskelinen M., Kataja V., Mannermaa A. (2010). *ST14* Gene Variant and Decreased Matriptase Protein Expression Predict Poor Breast Cancer Survival. Cancer Epidemiol. Biomark. Prev..

[48] Nilubol N., Boufraqech M., Zhang L., Kebebew E. (2014). Loss of *CPSF2* Expression Is Associated with Increased Thyroid Cancer Cellular Invasion and Cancer Stem Cell Population, and More Aggressive Disease. J. Clin. Endocrinol. Metab..

[49] Zhao C., Xu Z., Que H., Zhang K., Wang F., Tan R., Fan C. (2024). ASB1 Inhibits Prostate Cancer Progression by Destabilizing CHCHD3 via K48-Linked Ubiquitination. Am. J. Cancer Res..

[50] Khan S.U., Khan I.M., Khan M.U., Ud Din M.A., Khan M.Z., Khan N.M., Liu Y. (2023). Role of LGMN in Tumor Development and Its Progression and Connection with the Tumor Microenvironment. Front. Mol. Biosci..

[51] Bao L., Zhang Y., Wang J., Wang H., Dong N., Su X., Xu M., Wang X. (2016). Variations of Chromosome 2 Gene Expressions among Patients with Lung Cancer or Non-Cancer. Cell Biol. Toxicol..

[52] Zhang J., Huang J.Y., Chen Y.N., Yuan F., Zhang H., Yan F.H., Wang M.J., Wang G., Su M., Lu G. (2015). Whole Genome and Transcriptome Sequencing of Matched Primary and Peritoneal Metastatic Gastric Carcinoma. Sci. Rep..

[53] Liu L., Luo C., Luo Y., Chen L., Liu Y., Wang Y., Han J., Zhang Y., Wei N., Xie Z. (2018). MRPL33 and Its Splicing Regulator HnRNPK Are Required for Mitochondria Function and Implicated in Tumor Progression. Oncogene.

[54] Chodary Khameneh S., Razi S., Shamdani S., Uzan G., Naserian S. (2022). Weighted Correlation Network Analysis Revealed Novel Long Non-Coding RNAs for Colorectal Cancer. Sci. Rep..

[55] Li M., Chen R., Ji B., Fan C., Wang G., Yue C., Jin G. (2019). Impact of GTF2H1 and RAD54L2 Gene Polymorphisms on the Non-Small Cell Lung Cancer with Platinum Chemotherapy in High Altitude Areas 2019. Preprint.

[56] Ghate N.B., Kim S., Shin Y., Kim J., Doche M., Valena S., Situ A., Kim S., Rhie S.K., Lenz H.-J. (2023). Phosphorylation and Stabilization of EZH2 by DCAF1/VprBP Trigger Aberrant Gene Silencing in Colon Cancer. Nat. Commun..

[57] Chen W., Cheng L., Xu L., Qian Q., Zhu Y. (2019). Bioinformatics Analysis of Prognostic Value of *TRIM13* Gene in Breast Cancer. Biosci. Rep..

[58] Montero-Calle A., Jiménez de Ocaña S., Benavente-Naranjo R., Rejas-González R., Bartolomé R.A., Martínez-Useros J., Sanz R., Dziaková J., Fernández-Aceñero M.J., Mendiola M. (2023). Functional Proteomics Characterization of the Role of SPRYD7 in Colorectal Cancer Progression and Metastasis. Cells.

[59] Liu Y.-J., Yin S.-Y., Zeng S.-H., Hu Y.-D., Wang M.-Q., Huang P., Li J.-P. (2021). Prognostic Value of LHFPL Tetraspan Subfamily Member 6 (LHFPL6) in Gastric Cancer: A Study Based on Bioinformatics Analysis and Experimental Validation. Pharmgenomics Pers. Med..

[60] Karlsson E.K., Sigurdsson S., Ivansson E., Thomas R., Elvers I., Wright J., Howald C., Tonomura N., Perloski M., Swofford R. (2013). Genome-Wide Analyses Implicate 33 Loci in Heritable Dog Osteosarcoma, Including Regulatory Variants near CDKN2A/B. Genome Biol..

[61] Arias K.D., Fernández I., Traoré A., Goyache F. (2024). West African Cattle Share Non-Random Heterozygosity-Rich Region Islands Enriched on Adaptation-Related Genes despite Their Different Origins. Front. Anim. Sci..

[62] Cassandri M., Smirnov A., Novelli F., Pitolli C., Agostini M., Malewicz M., Melino G., Raschellà G. (2017). Zinc-Finger Proteins in Health and Disease. Cell Death Discov..

[63] Clements D.N., Carter S.D., Innes J.F., Ollier W.E.R. (2006). Genetic Basis of Secondary Osteoarthritis in Dogs with Joint Dysplasia. Am. J. Vet. Res..

[64] Imai K., Mizukami T., Takahashi T., Watanabe T., Abiko Y. (2011). Osteomodulin Gene Expression in Beagle Dog Mandible by Beta-Tricalcium Phosphate. Int. J. Oral-Med. Sci..

[65] Deckx S., Heymans S., Papageorgiou A. (2016). The Diverse Functions of Osteoglycin: A Deceitful Dwarf, or a Master Regulator of Disease?. FASEB J..

[66] Dubail J., Apte S.S. (2015). Insights on ADAMTS Proteases and ADAMTS-like Proteins from Mammalian Genetics. Matrix Biol..

[67] Vallet M. (2017). Study of RIN3: A Susceptibility Gene for Paget’s Disease of Bone. Ph.D. Thesis.

[68] Xu Y., Yang X.-L., Yang X.-L., Ren Y.-R., Zhuang X.-Y., Zhang L., Zhang X.-F. (2020). Functional Annotations of Single-Nucleotide Polymorphism (SNP)-Based and Gene-Based Genome-Wide Association Studies Show Genes Affecting Keratitis Susceptibility. Med. Sci. Monit..

[69] Kiener S., Wiener D.J., Hopke K., Diesel A.B., Jagannathan V., Mauldin E.A., Casal M.L., Leeb T. (2022). *ABHD5* Frameshift Deletion in Golden Retrievers with Ichthyosis. G3 Genes|Genomes|Genet..

[70] Shukla G.C., Padgett R.A. (2004). U4 Small Nuclear RNA Can Function in Both the Major and Minor Spliceosomes. Proc. Natl. Acad. Sci. USA.

[71] Lekholm E., Perland E., Eriksson M.M., Hellsten S.V., Lindberg F.A., Rostami J., Fredriksson R. (2017). Putative Membrane-Bound Transporters MFSD14A and MFSD14B Are Neuronal and Affected by Nutrient Availability. Front. Mol. Neurosci..

[72] Rolhion N., Furniss R.C.D., Grabe G., Ryan A., Liu M., Matthews S.A., Holden D.W. (2016). Inhibition of Nuclear Transport of NF-ĸB P65 by the Salmonella Type III Secretion System Effector SpvD. PLoS Pathog..

[73] Yu Y., Liu L., Windig J., Bosse M., Groenen M.A.M., Crooijmans R.P.M.A. (2022). Unique Genetic Signature and Selection Footprints in Dutch Population of German Longhaired Pointer Dogs. Anim. Genet..

[74] Perfilyeva A., Bespalova K., Bespalov S., Begmanova M., Kuzovleva Y., Vishnyakova O., Nazarenko I., Abylkassymova G., Perfilyeva Y., Plakhov K. (2023). Homozygosity Mapping in the Kazakh National Dog Breed Tazy. Sci. Rep..

[75] Mastrangelo S., Biscarini F., Riggio S., Ragatzu M., Spaterna A., Cendron F., Ciampolini R. (2024). Genome-Wide Association Study for Morphological and Hunting-Behavior Traits in Braque Français Type Pyrénées Dogs: A Preliminary Study. Vet. J..

[76] Hedrick P.W. (2007). Balancing Selection. Curr. Biol..

[77] Wang G., Cheng L., Fan R., Irwin D.M., Tang S., Peng J., Zhang Y. (2013). Signature of Balancing Selection at the MC1R Gene in Kunming Dog Populations. PLoS ONE.

[78] Croze M., Wollstein A., Božičević V., Živković D., Stephan W., Hutter S. (2017). A Genome-Wide Scan for Genes under Balancing Selection in Drosophila Melanogaster. BMC Evol. Biol..

[79] Tennessen J.A., Blouin M.S. (2008). Balancing Selection at a Frog Antimicrobial Peptide Locus: Fluctuating Immune Effector Alleles?. Mol. Biol. Evol..

[80] Moeller A.H. (2023). Metagenomic Signatures of Balancing Selection in the Human Gut. Mol. Ecol..

[81] Tetteh K.K.A., Stewart L.B., Ochola L.I., Amambua-Ngwa A., Thomas A.W., Marsh K., Weedall G.D., Conway D.J. (2009). Prospective Identification of Malaria Parasite Genes under Balancing Selection. PLoS ONE.

[82] Kang J., Xiang X., Chen X., Jiang J., Zhang Y., Li L., Tang J. (2023). Angiogenesis-Related Gene Signatures Reveal the Prognosis of Cervical Cancer Based on Single Cell Sequencing and Co-Expression Network Analysis. Front. Cell Dev. Biol..

[83] Yoh T., Hatano E., Kasai Y., Fuji H., Nishi K., Toriguchi K., Sueoka H., Ohno M., Seo S., Iwaisako K. (2019). Serum Nardilysin, a Surrogate Marker for Epithelial–Mesenchymal Transition, Predicts Prognosis of Intrahepatic Cholangiocarcinoma after Surgical Resection. Clin. Cancer Res..

[84] Xiong W., Yang S., Zhang W., Chen Y., Wang F. (2019). MiR-761 Inhibits Colorectal Cancer Cell Proliferation and Invasion through Targeting HDAC1. Pharmazie.

[85] Tsyba L., Nikolaienko O., Dergai O., Dergai M., Novokhatska O., Skrypkina I., Rynditch A. (2011). Intersectin Multidomain Adaptor Proteins: Regulation of Functional Diversity. Gene.

[86] Qin L., Wu Y.-L., Toneff M.J., Li D., Liao L., Gao X., Bane F.T., Tien J.C.-Y., Xu Y., Feng Z. (2014). NCOA1 Directly Targets *M-CSF1* Expression to Promote Breast Cancer Metastasis. Cancer Res..

[87] Klopfleisch R., Lenze D., Hummel M., Gruber A.D. (2011). The Metastatic Cascade Is Reflected in the Transcriptome of Metastatic Canine Mammary Carcinomas. Vet. J..

[88] Li Y., Xu J., Xiong H., Ma Z., Wang Z., Kipreos E.T., Dalton S., Zhao S. (2014). Cancer Driver Candidate Genes AVL9, DENND5A and NUPL1 Contribute to MDCK Cystogenesis. Oncoscience.

[89] Yao H., Zhang S., Xie H., Fan Y., Miao M., Zhu R., Yuan L., Gu M., You Y., You B. (2023). RCN2 Promotes Nasopharyngeal Carcinoma Progression by Curbing Calcium Flow and Mitochondrial Apoptosis. Cell. Oncol..

[90] Janssen W.J.M., Grobarova V., Leleux J., Jongeneel L., van Gijn M., van Montfrans J.M., Boes M. (2018). Proline-Serine-Threonine Phosphatase Interacting Protein 1 (PSTPIP1) Controls Immune Synapse Stability in Human T Cells. J. Allergy Clin. Immunol..

[91] Yang X., Liu G., Wang Q., Gao X., Xia T., Zhao C., Dou H., Zhang H. (2021). Comparative Transcriptome Provides Insights into the Selection Adaptation between Wild and Farmed Foxes. Ecol. Evol..

[92] Sharkia R., Zalan A., Kessel A., Al-Shareef W., Zahalka H., Hengel H., Schöls L., Azem A., Mahajnah M. (2024). SCAPER-Related Autosomal Recessive Retinitis Pigmentosa with Intellectual Disability: Confirming and Extending the Phenotypic Spectrum and Bioinformatics Analyses. Genes.

[93] Short A.D., Boag A., Catchpole B., Kennedy L.J., Massey J., Rothwell S., Husebye E., Ollier B. (2013). A Candidate Gene Analysis of Canine Hypoadrenocorticism in 3 Dog Breeds. J. Hered..

