# Supplementary material for: Heterozygosity-Rich Regions in Canine Genome: Can They Serve as Indicators of Balancing Selection?
Source: Animals (Basel). 2025 Feb 19;15(4):612. doi: 10.3390/ani15040612 (PMC11851536; doi:10.3390/ani15040612)
Supplement: Supplementary file 1 [file animals-15-00612-s001.zip › animals-3454237-supplementary.pdf]

Heterozygosity-Rich Regions in Canine Genome: Can They Serve as Indicators of Balancing Selection?

Adrián Halvoník [1,*], Nina Moravčíková [1,*], Luboš Vostrý [2], Hana Vostra-Vydrova [2], Gábor Mészáros [3], Eymen Demir [4], Monika Chalupková [1] and Radovan Kasarda [1]

Supplementary Table S1. List of examined breeds with their abbreviation, number of genotyped individuals, country of origin, category based on the degree of genetic distance, and FCI group and section.

Breed name and abbreviation	No. of genotyped animals	FCI group	FCI section	Genetic distance	Country of origin
Australian Shepherd (AS)	20	1	1	10	USA
Basenji (BA)	30	5	6	11	Central Africa
Basset Hound (BH)	20	6	1	4	Great Britain
Beagle (BE)	31	6	1	4	Great Britain
Belgian Tervuren (BT)	20	1	1	11	Belgium
Bernese Mountain Dog (BM)	34	2	3	9	Switzerland
Bichon Frise (BF)	22	9	1	3	France
Border Collie (BC)	67	1	1	10	Great Britain
Border Terrier (BR)	34	3	1	7	Great Britain
Boston Terrier (BS)	21	9	11	9	USA
Boxer (BO)	139	2	2	9	Germany
Bull Terrier (BL)	39	3	3	9	Great Britain
Cairn Terrier (CT)	98	3	2	7	Great Britain
Cavalier King Charles Spaniel (CK)	55	9	7	5	Great Britain
Cocker Spaniel (CS)	56	8	2	5	Great Britain
Czechoslovakian wolfdog (CW)	30	1	1	11	Slovakia
Dachshund (DA)	40	4	-	4	Germany
Doberman Pinscher (DP)	48	2	1	8	Germany
English Bulldog (EB)	25	2	2	9	Great Britain
English Cocker Spaniel (EC)	27	2	8	5	Great Britain
English Setter (ES)	97	7	2	6	Great Britain
English Springer Spaniel (EN)	106	8	2	5	Great Britain
French Bulldog (FB)	27	9	11	9	France
German Shepherd Dog (GD)	284	1	1	11	Germany
Golden Retriever (GR)	304	8	1	10	Great Britain

Gordon Setter (GS)	43	7	2	6	Great Britain
Greyhound (GH)	22	10	3	9	Great Britain
Havanese (HV)	44	9	1	3	Mediterranean region
Irish Wolfhound (IW)	264	10	2	9	Ireland
Jack Russell Terrier (JR)	37	3	2	7	Great Britain
Labrador Retriever (LR)	721	8	1	10	Great Britain
Maltese (ML)	85	9	1	3	Italy
Mastiff (MA)	22	2	2	9	Great Britain
Miniature Schnauzer (MS)	60	2	1	1	Germany
Newfoundland (NF)	106	2	2	10	Canada
Norfolk Terrier (NT)	21	3	2	7	Great Britain
Nova Scotia Duck Tolling Retriever (NR)	31	8	1	10	Canada
Papillon (PP)	32	9	9	2	Belgium
Poodle (PO)	50	9	2	3	France
Rottweiler (RT)	96	8	2	9	Germany
Saint Bernard (SB)	27	2	2	9	Switzerland
Slovakian Chuvach (SE)	22	1	1	11	Slovakia
Schipperke (SS)	26	1	1	2	Belgium
Scottish Terrier (ST)	27	3	2	7	Great Britain
Shetland Sheepdog (SP)	26	1	1	10	Great Britain
Shih Tzu (SC)	43	9	5	11	China
Tibetan Spaniel (TS)	31	9	5	11	China
Vizsla (VI)	93	7	1	6	Hungary
Weimaraner (WE)	36	7	1	6	Germany
Yorkshire Terrier (YT)	211	3	4	7	Great Britain

Supplementary Table S2. Information about HRRs, HRRIs, and observed heterozygosity per each autosomal chromosome with standard errors in brackets.

CHR	% of CHR covered by HRRs	No. of HRRs	No. of HRRIs	Average length of HRRI	Average no. of SNPs in HRRIs	Observed heterozygosity
1	0.058 (0.002)	4173	27	286.574 (20.103)	22.259 (1.257)	0.252 (0.001)
2	0.037 (0.001)	2652	12	248.414 (23.383)	20.833 (1.714)	0.250 (0.001)
3	0.049 (0.001)	3534	24	279.428 (36.818)	20.333 (2.108)	0.249 (0.001)
4	0.033 (0.001)	2390	5	285.988 (32.078)	20.400 (1.990)	0.248 (0.001)

5	0.039 (0.002)	2818	23	193.184 (18.034)	15.739 (1.296)	0.253 (0.001)
6	0.035 (0.001)	2591	18	216.16 (26.163)	18.111 (1.833)	0.249 (0.001)
7	0.033 (0.001)	2446	14	195.286 (26.543)	16.714 (2.042)	0.251 (0.001)
8	0.032 (0.001)	2322	11	253.607 (25.705)	20.727 (1.727)	0.263 (0.001)
9	0.022 (0.001)	1547	11	264.903 (31.878)	16.818 (1.916)	0.246 (0.002)
10	0.028 (0.001)	2041	10	328.887 (43.296)	24.600 (4.298)	0.250 (0.002)
11	0.021 (0.001)	1593	12	324.334 (26.760)	23.250 (1.366)	0.251 (0.002)
12	0.037 (0.001)	2589	20	268.408 (28.185)	18.850 (1.74)	0.250 (0.001)
13	0.042 (0.001)	2992	21	257.556 (32.752)	17.952 (1.963)	0.258 (0.001)
14	0.030 (0.001)	2175	20	269.232 (28.509)	22.950 (2.287)	0.251 (0.002)
15	0.023 (0.001)	1623	0	-	-	0.251 (0.002)
16	0.021 (0.001)	1490	9	246.512 (61.204)	18.889 (3.518)	0.253 (0.002)
17	0.039 (0.002)	2786	29	244.592 (23.532)	20.103 (1.807)	0.258 (0.001)
18	0.028 (0.001)	1987	18	262.251 (30.841)	19.111 (2.045)	0.253 (0.002)
19	0.034 (0.001)	2467	13	259.202 (42.049)	20.615 (3.018)	0.264 (0.002)
20	0.026 (0.001)	1872	17	296.498 (12.606)	22.706 (0.898)	0.246 (0.002)
21	0.022 (0.001)	1549	6	225.557 (46.110)	18.500 (3.384)	0.254 (0.002)
22	0.057 (0.002)	4054	56	243.512 (17.387)	18.857 (1.222)	0.252 (0.002)
23	0.029 (0.001)	2086	20	320.943 (23.794)	25.750 (1.769)	0.255 (0.002)
24	0.025 (0.001)	1804	15	260.112 (26.993)	18.867 (1.245)	0.25 (0.002)
25	0.027 (0.001)	1982	22	273.73 (29.455)	20.045 (2.027)	0.246 (0.002)
26	0.012 (0.001)	910	4	287.295 (2.154)	22 (0.577)	0.252 (0.002)
27	0.018 (0.001)	1362	3	327.443 (23.323)	23 (1.732)	0.259 (0.002)
28	0.025 (0.001)	1800	8	277.888 (47.282)	23 (2.994)	0.256 (0.002)
29	0.012 (0.001)	879	4	208.428 (34.346)	16 (2.483)	0.257 (0.002)
30	0.030 (0.001)	2120	14	208.501 (31.919)	15.571 (1.974)	0.258 (0.002)
31	0.010 (0.001)	706	5	254.964 (44.488)	21 (3.619)	0.258 (0.002)
32	0.009 (0.001)	643	0	-	-	0.254 (0.002)
33	0.011 (0.001)	785	8	367.216 (51.289)	22.250 (2.908)	0.261 (0.002)
34	0.025 (0.001)	1794	21	184.02 (24.979)	15.333 (1.830)	0.261 (0.002)
35	0.004 (0.000)	270	0	-	-	0.258 (0.002)
36	0.005 (0.001)	405	2	299.321 (11.992)	22.500 (2.500)	0.259 (0.002)
37	0.011 (0.001)	754	7	333.582 (34.954)	20.571 (1.192)	0.251 (0.002)
38	0.001 (0.000)	71	0	-	-	0.257 (0.002)

Supplementary Table S3. Information about Tajima's D statistics results per each autosomal chromosome with standard errors in brackets.

CHR	Mean D value	No. of selected windows	Average value of selected windows	Average no. of SNPs in selected windows
1	1.482 (0.053)	38	3.660 (0.013)	18.026 (0.349)
2	1.443 (0.049)	18	3.647 (0.026)	18.111 (0.471)
3	1.438 (0.052)	20	3.677 (0.024)	17.8 (0.49)
4	1.459 (0.063)	8	3.694 (0.022)	19.5 (0.655)
5	1.508 (0.050)	16	3.638 (0.016)	18.188 (0.66)
6	1.497 (0.043)	10	3.664 (0.019)	21.6 (0.884)
7	1.532 (0.042)	9	3.615 (0.023)	18.444 (0.944)
8	1.615 (0.045)	15	3.61 (0.033)	16.067 (0.396)
9	1.453 (0.063)	11	3.598 (0.025)	14.818 (0.685)
10	1.497 (0.040)	11	3.688 (0.044)	19 (0.944)
11	1.466 (0.055)	5	3.574 (0.044)	15 (1.761)
12	1.510 (0.059)	8	3.653 (0.036)	18.875 (0.441)
13	1.556 (0.041)	14	3.604 (0.024)	15.071 (0.659)
14	1.522 (0.040)	15	3.624 (0.025)	18.533 (0.542)
15	1.518 (0.064)	15	3.680 (0.023)	17.933 (0.419)
16	1.555 (0.053)	4	3.574 (0.059)	16 (1.08)
17	1.496 (0.059)	28	3.694 (0.018)	18.643 (0.659)
18	1.520 (0.047)	16	3.650 (0.016)	16.688 (0.445)
19	1.571 (0.060)	14	3.638 (0.023)	18.643 (0.723)
20	1.505 (0.055)	10	3.610 (0.019)	16.1 (0.657)
21	1.543 (0.048)	14	3.623 (0.016)	15.857 (0.977)
22	1.569 (0.053)	39	3.689 (0.015)	18.872 (0.418)
23	1.533 (0.055)	20	3.729 (0.029)	19.4 (0.494)
24	1.512 (0.057)	15	3.655 (0.022)	17.8 (0.718)
25	1.499 (0.054)	9	3.670 (0.032)	18 (0.408)
26	1.502 (0.046)	8	3.638 (0.036)	20.625 (1.322)
27	1.528 (0.046)	4	3.638 (0.059)	20 (1.225)
28	1.495 (0.047)	7	3.651 (0.036)	17.429 (0.685)
29	1.549 (0.050)	1	3.646 (-)	19 (-)
30	1.528 (0.056)	27	3.666 (0.014)	16.481 (0.516)
31	1.564 (0.059)	3	3.649 (0.032)	20.333 (1.856)
32	1.509 (0.056)	0	-	-
33	1.608 (0.045)	0	-	-

34	1.583 (0.056)	16	3.623 (0.018)	19.75 (1.055)
35	1.534 (0.063)	0	-	-
36	1.537 (0.061)	1	3.722 (-)	20 (-)
37	1.482 (0.055)	1	3.671 (-)	22 (-)
38	1.570 (0.046)	0	-	-

Supplementary Table S4. Descriptive statistics for HRRs per each breed and whole metapopulation with standard errors in brackets (standard error for % of genome covered by HRRs is not provided because all values were under three decimal points).

Breed	% of genome covered by HRRs	No. of HRRs	Mean length of HRRs (kb)	Mean observed heterozygosity	Longest HRR
AS	0.013	1722 (2.914)	331.368 (2.588)	0.304 (0.006)	1117.092
BA	0.004	606 (3.181)	319.175 (3.56)	0.215 (0.011)	863.048
BH	0.009	1268 (2.885)	318.873 (2.301)	0.257 (0.006)	887.044
BE	0.011	1481 (2.312)	323.843 (2.477)	0.286 (0.008)	878.050
BT	0.011	1495 (2.196)	328.807 (2.566)	0.264 (0.004)	996.883
BM	0.009	1273 (2.050)	326.287 (2.905)	0.231 (0.005)	1120.081
BF	0.011	1468 (2.900)	321.519 (2.34)	0.295 (0.006)	829.962
BC	0.010	1385 (2.687)	323.040 (2.574)	0.273 (0.007)	1101.423
BR	0.010	1407 (2.136)	323.166 (2.444)	0.226 (0.004)	865.663
BS	0.015	1993 (3.495)	321.716 (2.097)	0.275 (0.007)	892.654
BO	0.026	3523 (5.713)	329.539 (1.886)	0.227 (0.007)	1412.783
BL	0.008	1061 (3.147)	321.359 (2.638)	0.171 (0.001)	768.215
CT	0.005	1521 (1.308)	319.810 (3.659)	0.261 (0.004)	860.785
CK	0.011	1156 (2.581)	324.173 (2.277)	0.210 (0.006)	985.150
CS	0.009	1455 (2.100)	320.313 (2.733)	0.269 (0.005)	975.390
CW	0.011	775 (2.099)	312.052 (2.247)	0.239 (0.009)	865.663
DA	0.010	1353 (3.210)	331.527 (2.851)	0.263 (0.008)	975.390
DP	0.009	1252 (2.808)	331.406 (3.191)	0.217 (0.008)	962.156
EB	0.015	2043 (5.445)	319.536 (1.992)	0.253 (0.01)	887.044
EC	0.010	1326 (2.455)	324.459 (2.643)	0.246 (0.006)	975.390
ES	0.010	1330 (3.134)	319.791 (2.584)	0.255 (0.008)	975.071
EN	0.011	1442 (3.474)	325.363 (2.481)	0.261 (0.009)	875.368
FB	0.015	1969 (2.334)	325.833 (2.187)	0.269 (0.003)	975.071
GD	0.009	1184 (4.886)	322.045 (2.849)	0.232 (0.016)	958.487

GR	0.010	1443 (3.402)	320.460 (2.409)	0.250 (0.015)	887.044
GS	0.011	1548 (2.953)	320.728 (2.300)	0.275 (0.006)	865.663
GH	0.009	1231 (1.825)	317.101 (2.483)	0.240 (0.006)	952.374
HV	0.011	1431 (2.99)	328.765 (2.547)	0.282 (0.009)	785.837
IW	0.007	1002 (2.453)	321.650 (2.834)	0.178 (0.005)	896.314
JR	0.014	1880 (1.993)	326.219 (2.366)	0.328 (0.003)	1147.513
LR	0.012	1693 (4.095)	323.457 (2.367)	0.269 (0.014)	975.390
ML	0.009	1278 (2.806)	323.419 (2.471)	0.267 (0.011)	865.663
MA	0.011	1525 (2.116)	318.273 (2.108)	0.244 (0.003)	887.044
MS	0.009	1211 (2.308)	336.697 (3.034)	0.225 (0.009)	887.044
NF	0.011	1504 (2.959)	327.342 (2.447)	0.256 (0.007)	993.599
NT	0.010	1317 (2.652)	325.514 (2.603)	0.209 (0.006)	887.044
NR	0.012	1668 (2.214)	320.761 (2.298)	0.271 (0.003)	801.308
PP	0.011	1513 (2.593)	322.552 (2.472)	0.282 (0.005)	939.079
PO	0.014	1940 (4.617)	328.694 (2.146)	0.298 (0.009)	904.200
RT	0.010	1327 (2.896)	327.792 (2.976)	0.243 (0.008)	1057.998
SB	0.011	1496 (3.039)	320.661 (2.699)	0.256 (0.007)	1120.081
SE	0.011	1213 (2.796)	315.647 (2.34)	0.229 (0.007)	737.570
SS	0.009	1135 (2.190)	327.324 (2.54)	0.229 (0.006)	865.663
ST	0.008	1050 (2.162)	332.060 (2.774)	0.265 (0.006)	788.230
SP	0.008	1524 (2.116)	321.273 (2.932)	0.282 (0.004)	865.663
SC	0.010	1420 (2.415)	321.039 (2.319)	0.280 (0.003)	832.416
TS	0.008	1038 (1.955)	328.114 (2.936)	0.274 (0.004)	896.314
VI	0.011	1460 (2.204)	321.989 (2.278)	0.268 (0.007)	865.663
WE	0.010	1302 (3.233)	328.523 (2.759)	0.240 (0.008)	827.444
YT	0.010	1425 (2.124)	323.909 (2.401)	0.245 (0.004)	908.557
All breeds	0.011	72062 (0.764)	324.157 (0.357)	0.254 (0.001)	1412.783

Supplementary Table S5. Information about number, average length, and average number of SNPs of detected HRRIs per each breed and metapopulation with standard errors in brackets.

Breed	Number of HRRIs	No. of SNPs in HRRIs	Average no. of SNPs in HRRIs	Average length of HRRI
AS	9	159	17.667 (3.210)	0.222 (0.046)
BA	12	220	18.333 (3.210)	0.239 (0.026)

BH	10	212	21.200 (2.489)	0.288 (0.043)
BE	9	164	18.222 (2.676)	0.233 (0.036)
BT	7	153	21.857 (1.204)	0.278 (0.016)
BM	8	165	20.625 (1.647)	0.277 (0.026)
BF	17	294	17.294 (2.310)	0.232 (0.034)
BC	8	196	24.500 (2.155)	0.322 (0.033)
BR	10	184	18.400 (2.276)	0.245 (0.032)
BS	12	289	24.083 (3.159)	0.307 (0.051)
BO	11	190	17.273 (2.472)	0.221 (0.040)
BL	8	191	23.875 (2.216)	0.301 (0.037)
CT	17	394	23.200 (1.766)	0.298 (0.027)
CK	13	193	14.882 (1.424)	0.198 (0.024)
CS	9	183	20.308 (1.424)	0.264 (0.036)
CW	20	391	19.556 (2.534)	0.266 (0.039)
DA	8	210	26.250 (2.730)	0.371 (0.035)
DP	9	160	17.778 (3.947)	0.224 (0.046)
EB	7	153	21.857 (2.492)	0.287 (0.049)
EC	12	252	21.000 (2.987)	0.275 (0.049)
ES	8	146	18.250 (2.776)	0.231 (0.046)
EN	11	205	18.636 (1.845)	0.271 (0.034)
FB	9	155	17.222 (3.733)	0.204 (0.046)
GD	15	291	19.400 (1.511)	0.247 (0.026)
GR	7	146	20.857 (2.586)	0.264 (0.030)
GS	8	156	19.500 (2.976)	0.241 (0.044)
GH	9	179	19.889 (1.798)	0.253 (0.028)
HV	11	201	18.273 (3.311)	0.248 (0.046)
IW	9	193	21.444 (2.959)	0.300 (0.048)
JR	9	162	18.000 (2.438)	0.217 (0.032)

LR	8	163	20.375 (6.296)	0.248 (0.078)
ML	13	269	20.692 (1.635)	0.282 (0.022)
MA	10	151	15.100 (1.524)	0.179 (0.019)
MS	9	183	20.333 (2.687)	0.261 (0.030)
NF	8	145	18.125 (2.467)	0.236 (0.030)
NT	6	145	24.167 (3.331)	0.330 (0.066)
NR	11	248	22.545 (2.282)	0.310 (0.032)
PP	6	153	25.500 (2.895)	0.327 (0.045)
PO	12	222	18.500 (3.862)	0.246 (0.069)
RT	9	175	19.444 (0.915)	0.253 (0.024)
SB	7	164	23.429 (1.307)	0.338 (0.023)
SE	9	154	17.091 (1.904)	0.223 (0.033)
SS	8	150	18.778 (2.320)	0.260 (0.041)
ST	10	235	23.500 (3.723)	0.314 (0.045)
SP	11	215	19.500 (2.688)	0.266 (0.042)
SC	12	221	18.417 (2.334)	0.231 (0.035)
TS	11	246	22.364 (1.410)	0.301 (0.021)
VI	11	150	13.636 (2.660)	0.169 (0.037)
WE	15	348	23.200 (2.685)	0.288 (0.033)
YT	11	182	16.545 (2.560)	0.206 (0.036)
All breeds	509	4982	19.819 (0.372)	0.259 (0.006)

Supplementary Table S6. Information about Tajima's D statistics results per each breed and whole metapopulation with standard errors in brackets.

Breed	Average whole-genome D value	Average no. of SNPs per sliding window	Average D value of selected windows	Average no. of SNPs in selected windows	Total no. of SNPs in selected windows

AS	1.370 (0.009)	16.320 (0.050)	3.721 (0.026)	18.556 (1.271)	167
BA	0.565 (0.010)	16.320 (0.050)	3.492 (0.044)	18.000 (1.106)	162
BH	1.241 (0.010)	16.320 (0.050)	3.617 (0.023)	17.000 (0.799)	153
BE	1.437 (0.009)	16.320 (0.050)	3.601 (0.032)	15.778 (0.760)	142
BT	1.332 (0.011)	16.320 (0.050)	3.687 (0.015)	17.444 (1.002)	157
BM	1.066 (0.012)	16.320 (0.050)	3.671 (0.032)	19.333 (0.707)	174
BF	1.433 (0.009)	16.320 (0.050)	3.628 (0.024)	17.444 (1.082)	157
BC	1.291 (0.010)	16.320 (0.050)	3.603 (0.025)	16.556 (0.835)	149
BR	1.268 (0.012)	16.320 (0.050)	3.697 (0.011)	18.556 (0.766)	167
BS	1.296 (0.011)	16.320 (0.050)	3.627 (0.016)	17.778 (1.051)	160
BO	1.175 (0.013)	16.320 (0.050)	3.807 (0.022)	20.111 (0.841)	181
BL	0.309 (0.015)	16.320 (0.050)	3.574 (0.026)	17.333 (1.130)	156
CT	1.157 (0.011)	16.320 (0.050)	3.533 (0.021)	16.444 (0.729)	148
CK	1.364 (0.010)	16.320 (0.050)	3.720 (0.015)	18.111 (1.136)	163
CS	1.235 (0.013)	16.320 (0.050)	3.732 (0.019)	19.556 (0.709)	176
CW	1.119 (0.011)	16.320 (0.050)	3.644 (0.010)	17.778 (0.878)	160
DA	1.444 (0.009)	16.320 (0.050)	3.624 (0.022)	17.444 (1.002)	157
DP	1.054 (0.012)	16.320 (0.050)	3.739 (0.026)	17.778 (0.778)	160
EB	1.012 (0.012)	16.320 (0.050)	3.730 (0.015)	18.889 (1.033)	170
EC	1.263 (0.011)	16.320 (0.050)	3.677 (0.019)	17.667 (1.143)	159
ES	1.203 (0.010)	16.320 (0.050)	3.679 (0.029)	17.778 (0.813)	160
EN	1.495 (0.009)	16.320 (0.050)	3.681 (0.017)	17.000 (0.745)	153
FB	1.346 (0.011)	16.320 (0.050)	3.704 (0.023)	21.889 (0.824)	197
GD	1.087 (0.010)	16.320 (0.050)	3.544 (0.026)	18.000 (1.280)	162
GR	1.356 (0.009)	16.320 (0.050)	3.607 (0.026)	16.111 (0.904)	145
GS	1.435 (0.009)	16.320 (0.050)	3.516 (0.024)	17.111 (0.676)	154
GH	1.327 (0.011)	16.320 (0.050)	3.651 (0.020)	16.333 (0.882)	147
HV	1.419 (0.009)	16.320 (0.050)	3.626 (0.034)	18.778 (0.760)	169
IW	1.315 (0.013)	16.320 (0.050)	3.756 (0.012)	19.444 (0.915)	175
JR	1.592 (0.009)	16.320 (0.050)	3.620 (0.030)	16.667 (0.441)	150
LR	1.474 (0.009)	16.320 (0.050)	3.709 (0.030)	18.556 (0.852)	167
ML	1.377 (0.009)	16.320 (0.050)	3.643 (0.032)	17.556 (1.180)	158
MA	1.162 (0.012)	16.320 (0.050)	3.627 (0.015)	17.667 (0.943)	159
MS	0.951 (0.011)	16.320 (0.050)	3.701 (0.023)	17.778 (0.862)	160
NF	1.404 (0.010)	16.320 (0.050)	3.629 (0.021)	18.222 (1.267)	164
NT	1.139 (0.013)	16.320 (0.050)	3.693 (0.015)	19.000 (0.957)	171
NR	1.396 (0.010)	16.320 (0.050)	3.731 (0.028)	19.222 (0.683)	173
PP	1.455 (0.009)	16.320 (0.050)	3.665 (0.020)	16.778 (0.795)	151

PO	1.622 (0.009)	16.320 (0.050)	3.593 (0.021)	17.222 (0.722)	155
RT	1.112 (0.011)	16.320 (0.050)	3.725 (0.024)	18.667 (0.726)	168
SB	1.313 (0.010)	16.320 (0.050)	3.647 (0.023)	17.556 (1.094)	158
SE	1.426 (0.010)	16.320 (0.050)	3.618 (0.018)	17.444 (1.355)	157
SS	0.997 (0.012)	16.320 (0.050)	3.625 (0.017)	17.778 (0.983)	160
ST	1.119 (0.012)	16.320 (0.050)	3.758 (0.028)	18.556 (0.930)	167
SP	1.257 (0.010)	16.320 (0.050)	3.689 (0.033)	17.333 (0.833)	156
SC	1.520 (0.009)	16.320 (0.050)	3.589 (0.032)	18.111 (1.369)	163
TS	1.423 (0.010)	16.320 (0.050)	3.638 (0.016)	18.556 (0.801)	167
VI	1.443 (0.010)	16.320 (0.050)	3.652 (0.020)	18.000 (0.645)	162
WE	1.197 (0.011)	16.320 (0.050)	3.722 (0.034)	19.667 (1.190)	177
YT	1.359 (0.010)	16.320 (0.050)	3.614 (0.019)	18.889 (0.978)	170
All breeds	1.263 (0.002)	16.320 (0.007)	3.655 (0.095)	17.984 (2.895	8093

Supplementary Table S7. Results of gene enrichment analysis based on annotated genes within HRR islands detected in at least seven breeds in the same genomic region.

Term	Type	Genes	Fold Enrichment	*p*-value
GO:0005516~calmodulin binding	MF	*ENSCAFG00845018631, ENSCAFG00845018575, ENSCAFG00845020809, ENSCAFG00845018659, ENSCAFG00845018549*	12.84	0.001
KW-0378~Hydrolase	MF	*ENSCAFG00845009907, ENSCAFG00845019031, ENSCAFG00845006491, ENSCAFG00845006293, ENSCAFG00845008674, ENSCAFG00845009100, ENSCAFG00845021275, ENSCAFG00845009711, ENSCAFG00845008741, ENSCAFG00845006356*	2.66	0.006
GO:0051237~maintenance of RNA location	BP	*ENSCAFG00845009475*	225.48	0.009
GO:0016887~ATP hydrolysis activity	MF	*ENSCAFG00845019031, ENSCAFG00845020275, ENSCAFG00845009100, ENSCAFG00845009711, ENSCAFG00845008741*	5.72	0.011
GO:0031012~extracellular matrix	CC	*ENSCAFG00845006293, ENSCAFG00845024776, ENSCAFG00845024930, ENSCAFG00845024885*	8.33	0.012
GO:0062023~collagen-containing extracellular matrix	CC	*ENSCAFG00845025571, ENSCAFG00845006293, ENSCAFG00845008674, ENSCAFG00845006356*	8.28	0.012

GO:0000301~retrograde transport, vesicle recycling within Golgi	BP	*ENSCAFG00845024231, ENSCAFG00845022111*	112.74	0.017
GO:0035308~negative regulation of protein dephosphorylation	BP	*ENSCAFG00845009475*	90.19	0.021
KW-0645~Protease	MF	*ENSCAFG00845006491, ENSCAFG00845006293, ENSCAFG00845008674, ENSCAFG00845021275, ENSCAFG00845006356*	4.34	0.023
KW-0482~Metalloprotease	MF	*ENSCAFG00845006293, ENSCAFG00845008674, ENSCAFG00845006356*	11.54	0.025
GO:0035196~miRNA processing	BP	*ENSCAFG00845009475*	75.16	0.026
GO:0051457~maintenance of protein location in nucleus	BP	*ENSCAFG00845009223*	53.05	0.036
GO:0030198~extracellular matrix organization	BP	*ENSCAFG00845006293, ENSCAFG00845008674, ENSCAFG00845006356*	8.84	0.043
GO:0004865~protein serine/threonine phosphatase inhibitor activity	MF	*ENSCAFG00845009475*	42.00	0.046

BP = biological process; CC = cellular component; MF = molecular function.

Supplementary Table S8. Results of gene enrichment analysis based on annotated genes within signals of balancing selection detected in at least seven breeds in the same genomic region.

Term	Type	Genes	Fold Enrichment	*p*-value
GO:0005829~cytosol	CC	*ENSCAFG00845021857, ENSCAFG00845013496, ENSCAFG00845020085, ENSCAFG00845021372, ENSCAFG00845020170, ENSCAFG00845023394,*	2.730	0.004

		ENSCAFG00845020275, ENSCAFG00845024663, ENSCAFG00845022303, ENSCAFG00845023116		
GO:0031489~myosin V binding	MF	*ENSCAFG00845021135*	118.994	0.016
GO:0006904~vesicle docking involved in exocytosis	BP	*ENSCAFG00845021135*	94.937	0.020
GO:0017157~regulation of exocytosis	BP	*ENSCAFG00845021135*	64.312	0.029
GO:0009306~protein secretion	BP	*ENSCAFG00845021135*	37.617	0.049

BP = biological process; CC = cellular component; MF = molecular function.

Supplementary Table S9. List of all overlaps between HRRIs and signals of balancing selection

**Breed**	**CHR**	**HRRIs**		**Signals of balancing selection**	
		**Start**	**End**	**Start**	**End**
Australian Shepherd	22	1719379	2238632	2000000	2250000
Australian Shepherd	22	2264410	2399673	2250000	2500000
Australian Shepherd	22	2421940	2513263	2250000	2500000
Basenji	9	2004954	2255889	2000000	2250000
Basenji	22	3328253	3560590	3250000	3500000
Basenji	30	2585419	2756584	2750000	3000000
Basenji	30	2775667	2787549	2750000	3000000
Basset Hound	12	41877046	41914162	41750000	42000000
Basset Hound	22	6762346	7241794	7000000	7250000
Beagle	2	77903613	78191482	78000000	78250000
Belgian Tervuren	3	2348298	2602488	2500000	2750000
Bernese Mountain Dog	6	30488752	30641263	30500000	30750000
Bernese Mountain Dog	34	5391351	5575501	5500000	5750000
Bichon Frise	22	6788053	7216686	7000000	7250000
Bichon Frise	26	791595	1082621	1000000	1250000
Border Collie	14	3672303	3884041	3500000	3750000
Border Collie	14	3672303	3884041	3750000	4000000
Border Collie	18	2401012	2858609	2500000	2750000
Border Collie	18	2401012	2858609	2750000	3000000
Border Terrier	8	1823545	2102164	2000000	2250000

Border Terrier	17	18882940	19159876	19000000	19250000
Border Terrier	34	3366719	3626584	3500000	3750000
Boston Terrier	1	99594273	99915987	99750000	100000000
Boston Terrier	3	386718	682188	500000	750000
Boston Terrier	25	1599195	1918540	1750000	2000000
Boston Terrier	25	2268042	3010397	2500000	2750000
Boxer	27	23825888	24112228	24000000	24250000
Cairn Terrier	19	4767099	4948692	4750000	5000000
Cavalier King Charles Spaniel	2	77763637	78156996	78000000	78250000
Cavalier King Charles Spaniel	8	1774061	2124632	2000000	2250000
Cavalier King Charles Spaniel	9	2914011	3246506	3000000	3250000
Cocker Spaniel	20	14461218	14848529	14750000	15000000
Czechoslovakian wolfdog	13	1272226	1845505	1500000	1750000
Dachshund	13	4067434	4345039	4250000	4500000
Dachshund	22	2022950	2376165	2000000	2250000
Dachshund	22	50006780	50476642	50250000	50500000
Dachshund	28	14244202	14758462	14500000	14750000
English Bulldog	1	46959327	47042927	47000000	47250000
English Bulldog	1	98705220	98962840	98750000	99000000
English Bulldog	23	32780599	33296825	33000000	33250000
English Cocker Spaniel	2	3151017	3318983	3250000	3500000
English Setter	21	24621408	24640906	24500000	24750000
French Bulldog	6	45495451	45949718	45750000	46000000
French Bulldog	6	45983719	46022567	45750000	46000000
German Shepherd Dog	1	99483627	99915987	99750000	100000000
German Shepherd Dog	24	17661253	17907758	17750000	18000000
German Shepherd Dog	25	1599195	1907645	1750000	2000000
Golden Retriever	20	38184737	38497711	38250000	38500000
Golden Retriever	22	1889131	2238632	2000000	2250000
Gordon Setter	17	21887297	22263657	22000000	22250000
Gordon Setter	20	38219585	38519198	38500000	38750000
Greyhound	8	22563597	22904279	22750000	23000000
Havanese	1	99483627	99864950	99750000	100000000
Havanese	22	6874434	7007850	7000000	7250000
Havanese	23	2497591	3043486	2750000	3000000

Havanese	23	2497591	3043486	3000000	3250000
Irish Wolfhound	12	41599982	41779226	41750000	42000000
Irish Wolfhound	12	41828718	41877046	41750000	42000000
Labrador Retriever	14	2948414	3202559	3000000	3250000
Labrador Retriever	14	3232879	3929644	3000000	3250000
Labrador Retriever	14	3232879	3929644	3750000	4000000
Labrador Retriever	18	2524593	2858609	2750000	3000000
Labrador Retriever	22	1788707	2144778	2000000	2250000
Labrador Retriever	22	2180601	2238632	2000000	2250000
Maltese	13	1823801	2137889	2000000	2250000
Maltese	23	2620410	2987660	2750000	3000000
Mastiff	6	45749740	45949718	45750000	46000000
Mastiff	22	40402362	40567518	40500000	40750000
Miniature Schnauzer	5	3877304	4047823	4000000	4250000
Miniature Schnauzer	5	4089894	4259640	4000000	4250000
Miniature Schnauzer	17	18882940	19159876	19000000	19250000
Miniature Schnauzer	30	1484942	1833885	1750000	2000000
Nova Scotia Duck Tolling Retriever	21	24312988	24640906	24500000	24750000
Nova Scotia Duck Tolling Retriever	23	2640873	2987660	2750000	3000000
Papillon	1	60082153	60411738	60250000	60500000
Poodle	3	2285043	3197654	2500000	2750000
Poodle	3	2285043	3197654	2750000	3000000
Poodle	6	6533433	6897132	6750000	7000000
Rottweiler	3	49031034	49334630	49250000	49500000
Rottweiler	14	3751558	3945297	3750000	4000000
Rottweiler	22	28540165	28772080	28750000	29000000
Saint Bernard	10	4326189	4664700	4500000	4750000
Saint Bernard	17	21904651	22289388	22000000	22250000
Scottish Terrier	5	3877304	4199573	4000000	4250000
Scottish Terrier	7	45613579	45909278	45750000	46000000
Shetland Sheepdog	1	115012948	115273139	115250000	115500000
Shetland Sheepdog	10	19147970	19676707	19250000	19500000
Shetland Sheepdog	10	19147970	19676707	19500000	19750000
Shetland Sheepdog	34	18341784	18751035	18500000	18750000
Shih Tzu	3	2348298	2761663	2500000	2750000
Shih Tzu	3	2348298	2761663	2750000	3000000

Shih Tzu	18	2505760	2847593	2500000	2750000
Shih Tzu	18	2505760	2847593	2750000	3000000
Schipperke	4	24565947	24835581	24750000	25000000
Schipperke	23	29395995	29644654	29500000	29750000
Slovakian Chuvach	17	3801584	4117547	4000000	4250000
Tibetan Spaniel	1	99483627	99915987	99500000	99750000
Tibetan Spaniel	3	17362458	17645542	17500000	17750000
Tibetan Spaniel	20	2787700	3150898	3000000	3250000
Vizsla	22	5006729	5342898	5000000	5250000
Weimaraner	1	99538302	99915987	99750000	100000000
Weimaraner	3	60764954	61038500	61000000	61250000
Weimaraner	17	1374279	1716674	1500000	1750000
Weimaraner	17	3012133	3352889	3250000	3500000
Weimaraner	17	3774617	4012088	3750000	4000000
Weimaraner	23	2620410	3021121	2750000	3000000
Yorkshire Terrier	22	1892027	2238632	2000000	2250000
Yorkshire Terrier	25	2268042	2548504	2500000	2750000
Yorkshire Terrier	30	24896093	25082545	25000000	25250000

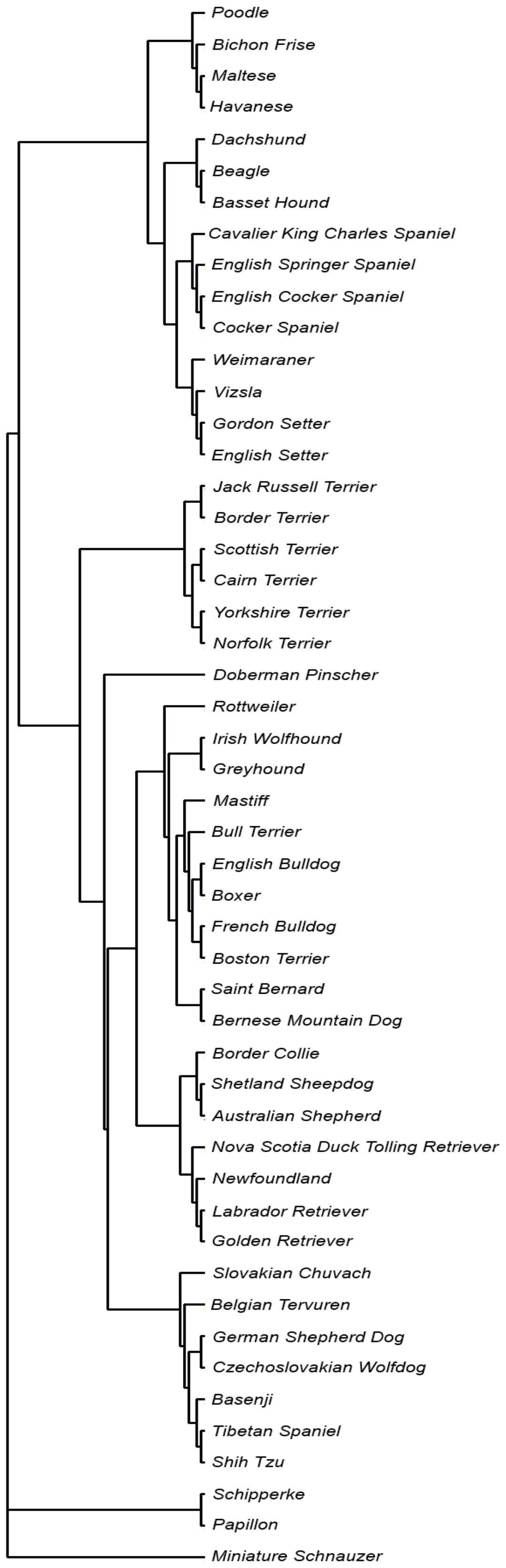

Supplementary Figure S1. Neighbour-joining tree constructed based Wright's F_{ST} matrix among studied breeds.
